# Supplementary figures and images for: ceRNAR: An R package for identification and analysis of ceRNA-miRNA triplets
Source: PLoS Comput Biol. 2022 Sep 9;18(9):e1010497. doi: 10.1371/journal.pcbi.1010497 (PMC9491567; doi:10.1371/journal.pcbi.1010497)

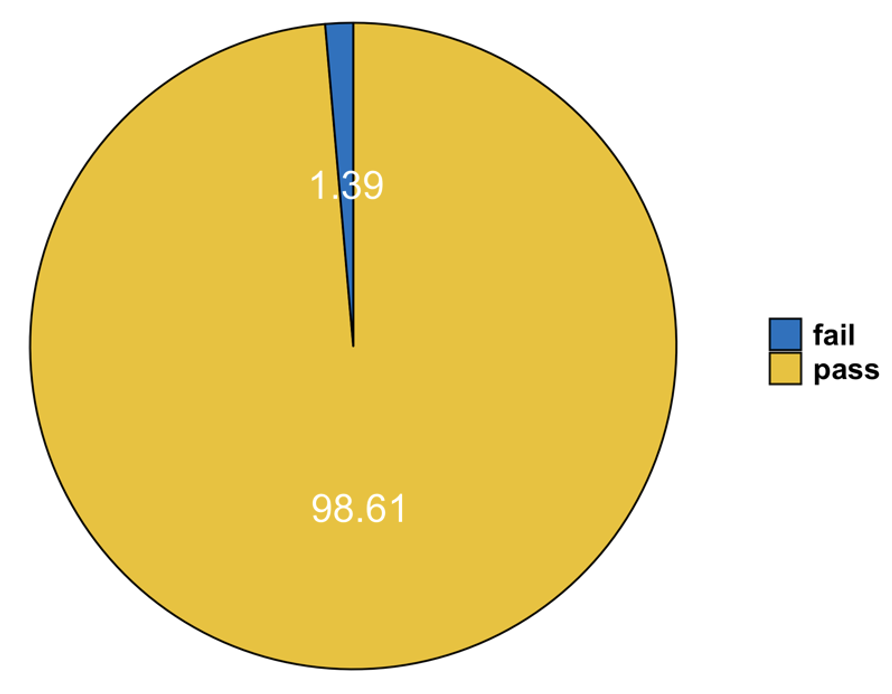

Supplement: S1 Fig — (TIFF) [file pcbi.1010497.s001.tiff]

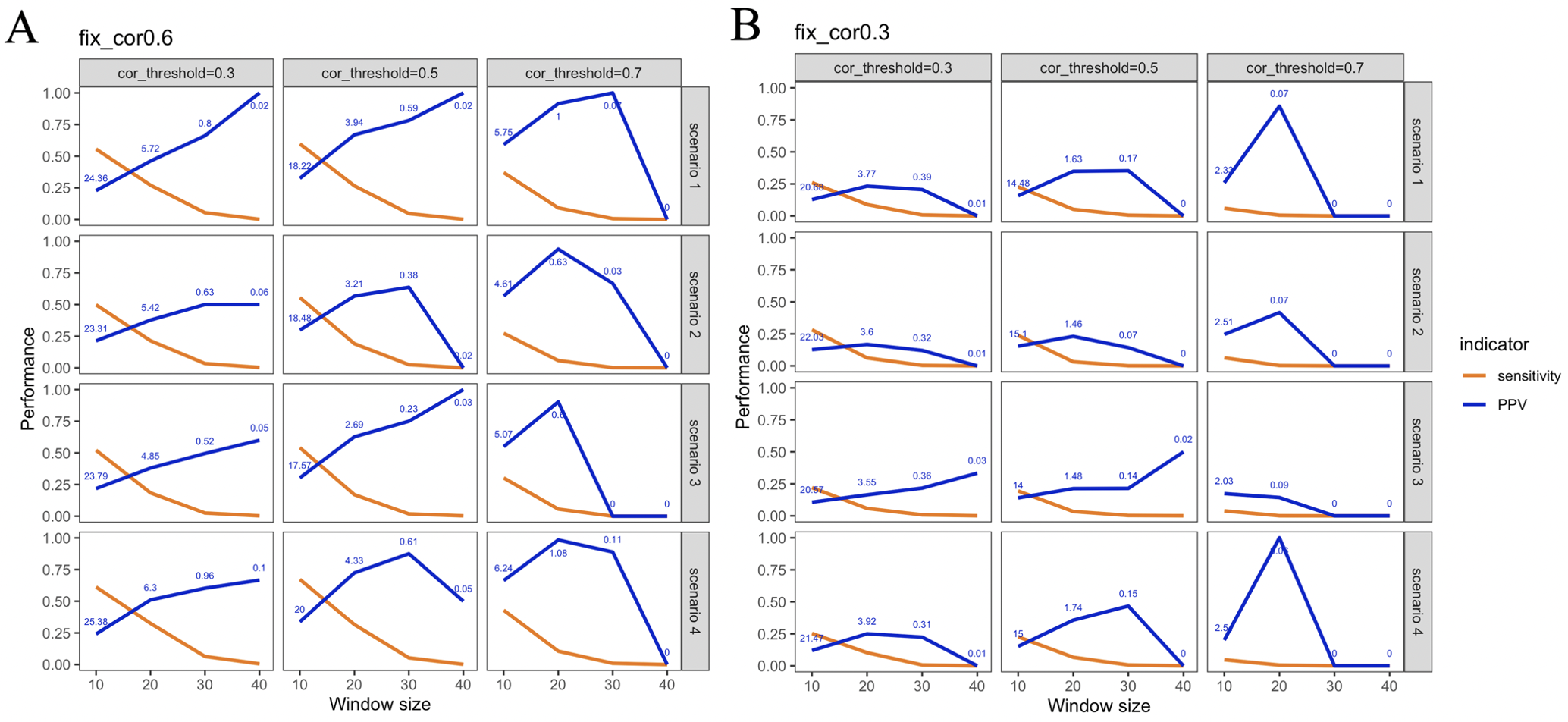

Supplement: S2 Fig — (A) Synthetic data of correlated genes were generated from multivariate normal distribution with a mean value of 0 and a covariance matrix whose entries are 0.6. (B) Synthetic data of correlated genes were generated from multivariate normal distribution with a mean value of 0 and a covariance matrix whose entries are 0.3. The numbers in blue represent the average number of identified ceRNA-miRNA triplets after 100 simulations. (TIFF) [file pcbi.1010497.s002.tiff]

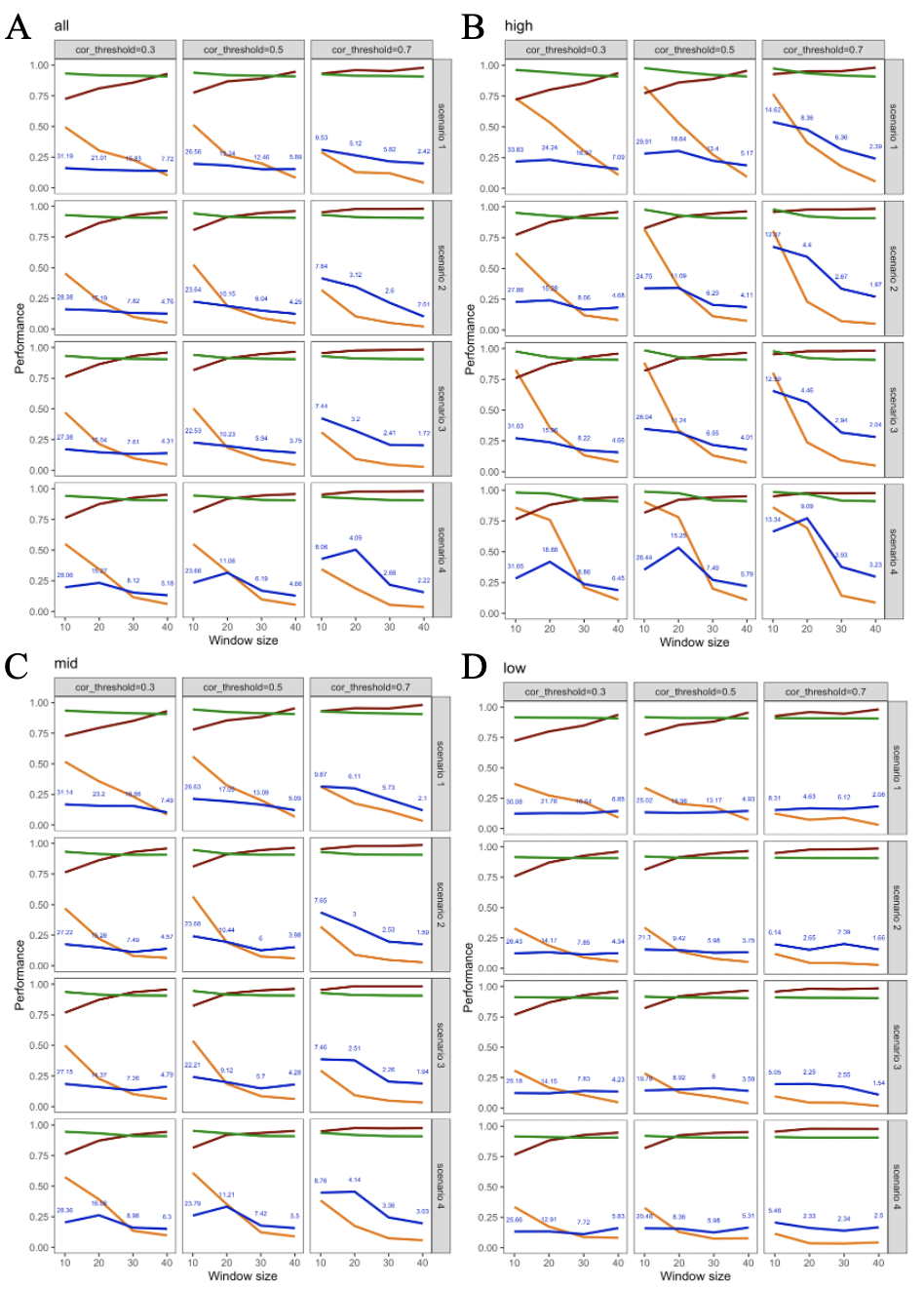

Supplement: S3 Fig — (A) From a covariance matrix with correlation values ranging from 0.3 to 0.9. (B) From a covariance matrix with correlation values ranging from 0.8 to 0.9. (C) From a covariance matrix with correlation values ranging from 0.5 to 0.7. (D) From a covariance matrix with correlation values ranging from 0.3 to 0.4. (TIFF) [file pcbi.1010497.s003.tiff]

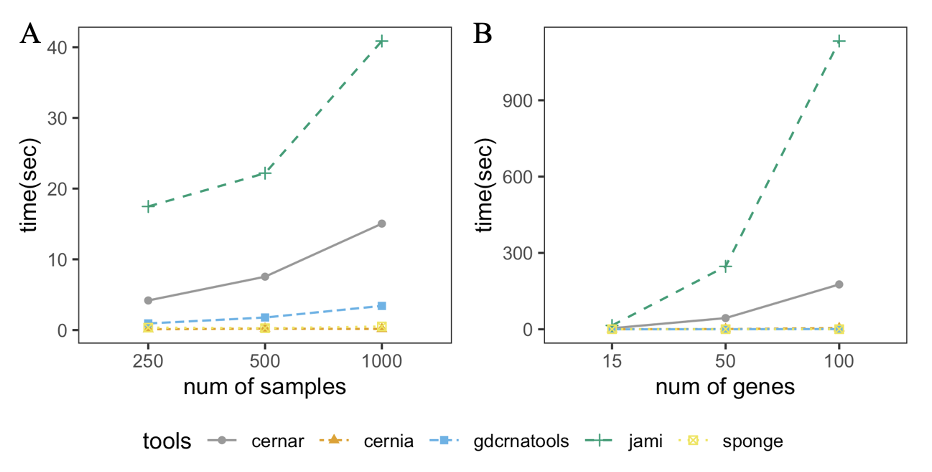

Supplement: S4 Fig — (A) Run time for different sample numbers on a fixed set of genes using real data. (B) Run time for different numbers of genes (i.e., different numbers of triplets) on a fixed number of samples using real data. (TIFF) [file pcbi.1010497.s004.tiff]

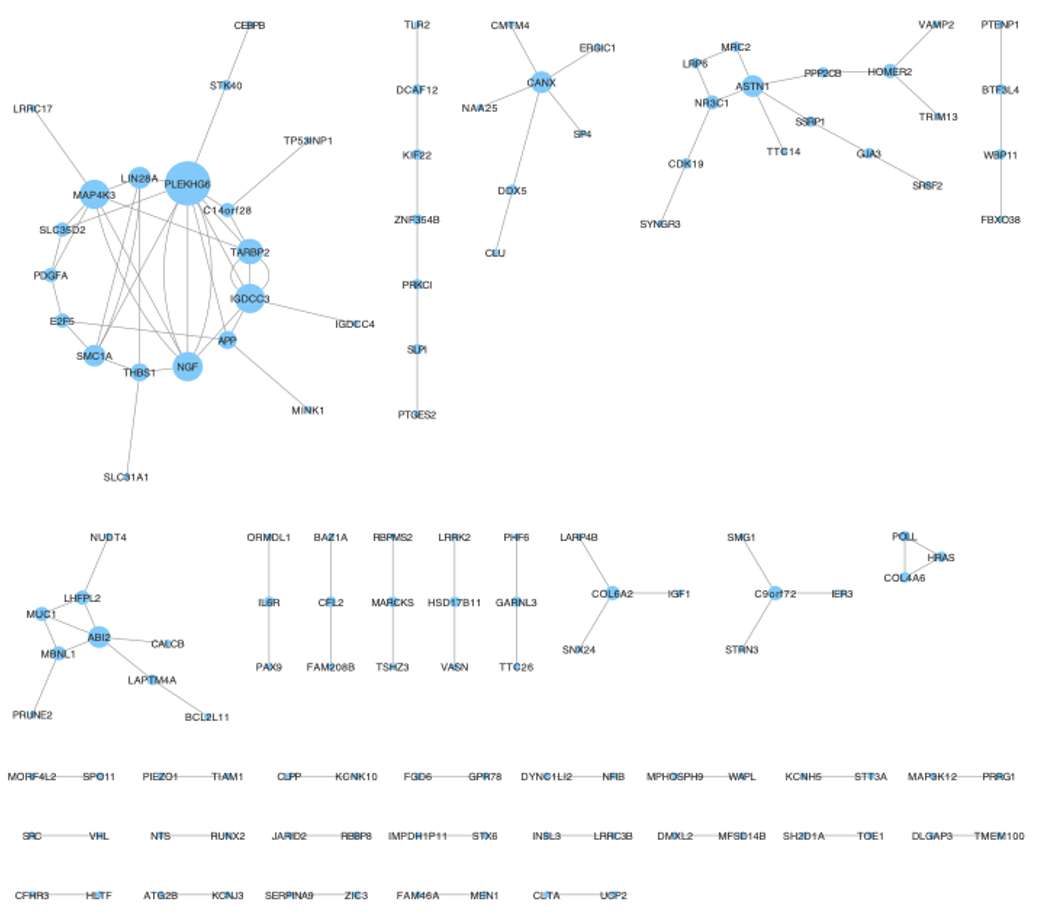

Supplement: S5 Fig — The size of each dot represents the number of bridged miRNAs per ceRNA. (TIFF) [file pcbi.1010497.s005.tiff]

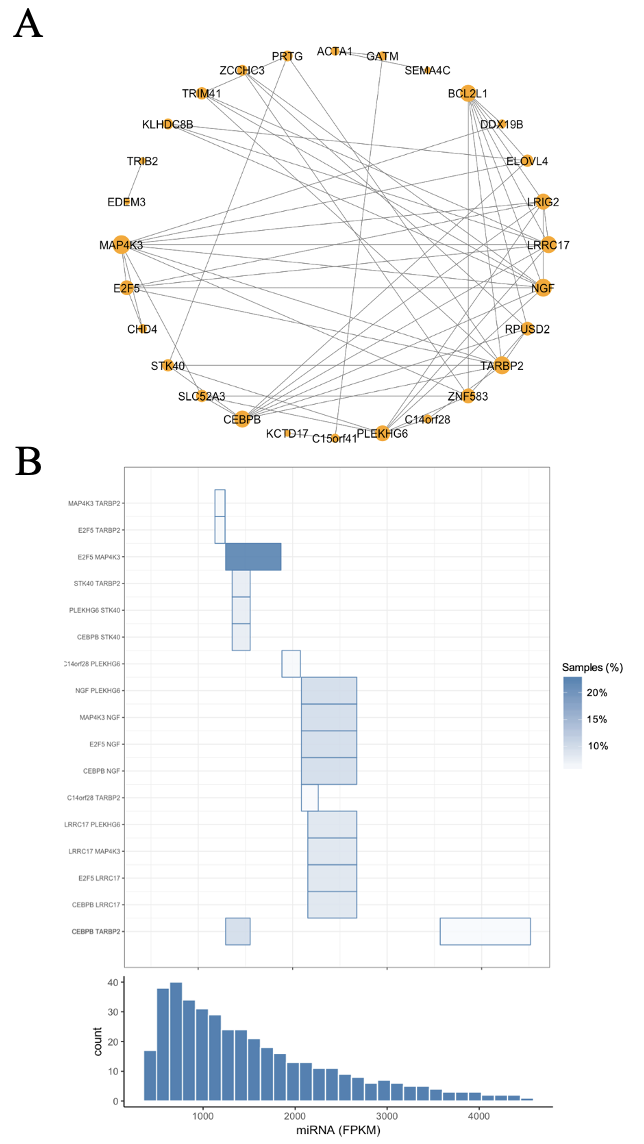

Supplement: S6 Fig — (A) The network of overlapping ceRNA pairs in both TCGA datasets. (B) The distribution of miRNA expression at which specific ceRNA interactions occur. The location of each rectangle indicates the miRNA expression value at which particular ceRNA pairs interact with miRNA has-let-17e-5p, and the depth of the color in each rectangle represents the number of samples that have such ceRNA-miRNA interaction. FPKM, fragments per kilobase million. (TIFF) [file pcbi.1010497.s006.tiff]

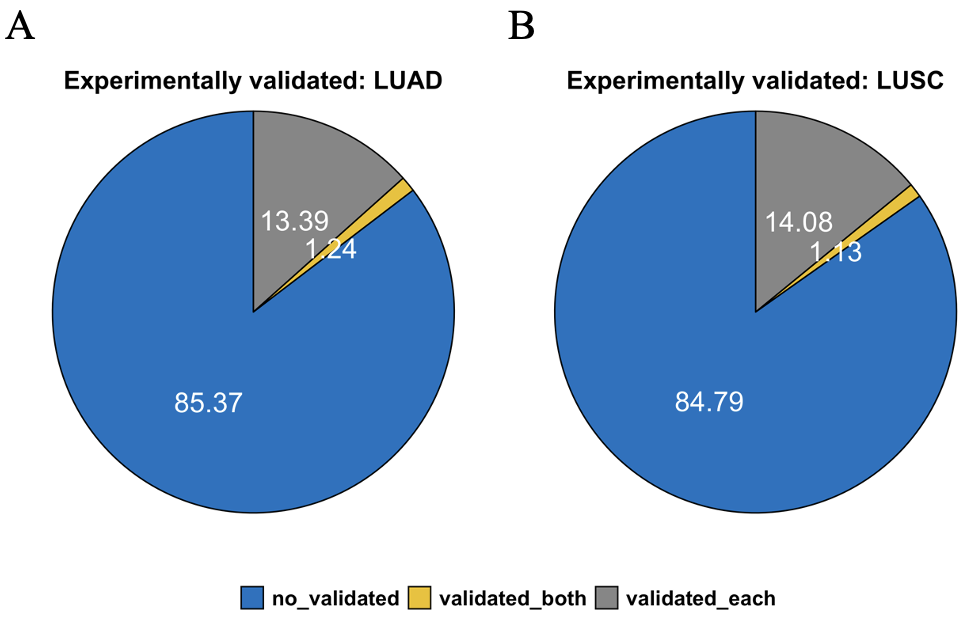

Supplement: S7 Fig — (A) TCGA-LUAD (The Cancer Genome Atlas Lung Adenocarcinoma). (B) TCGA-LUSC (The Cancer Genome Atlas Lung Squamous Cell Carcinoma). (TIFF) [file pcbi.1010497.s007.tiff]

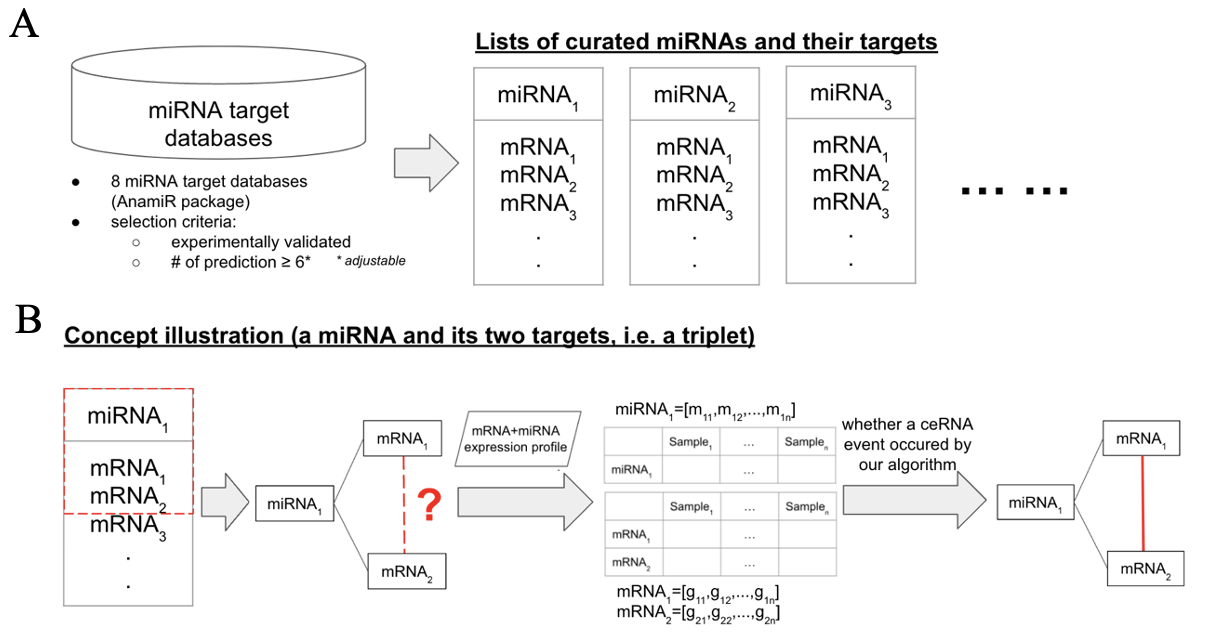

Supplement: S8 Fig — (A) Lists of curated miRNAs and their targets are verified from nine miRNA target databases based on the AnamiR package and selected by being either experimentally validated or present in over half of the prediction databases. (B) Our algorithm iteratively evaluates whether each mRNA pair is a potential ceRNA event in each miRNA-target list. (TIFF) [file pcbi.1010497.s008.tiff]

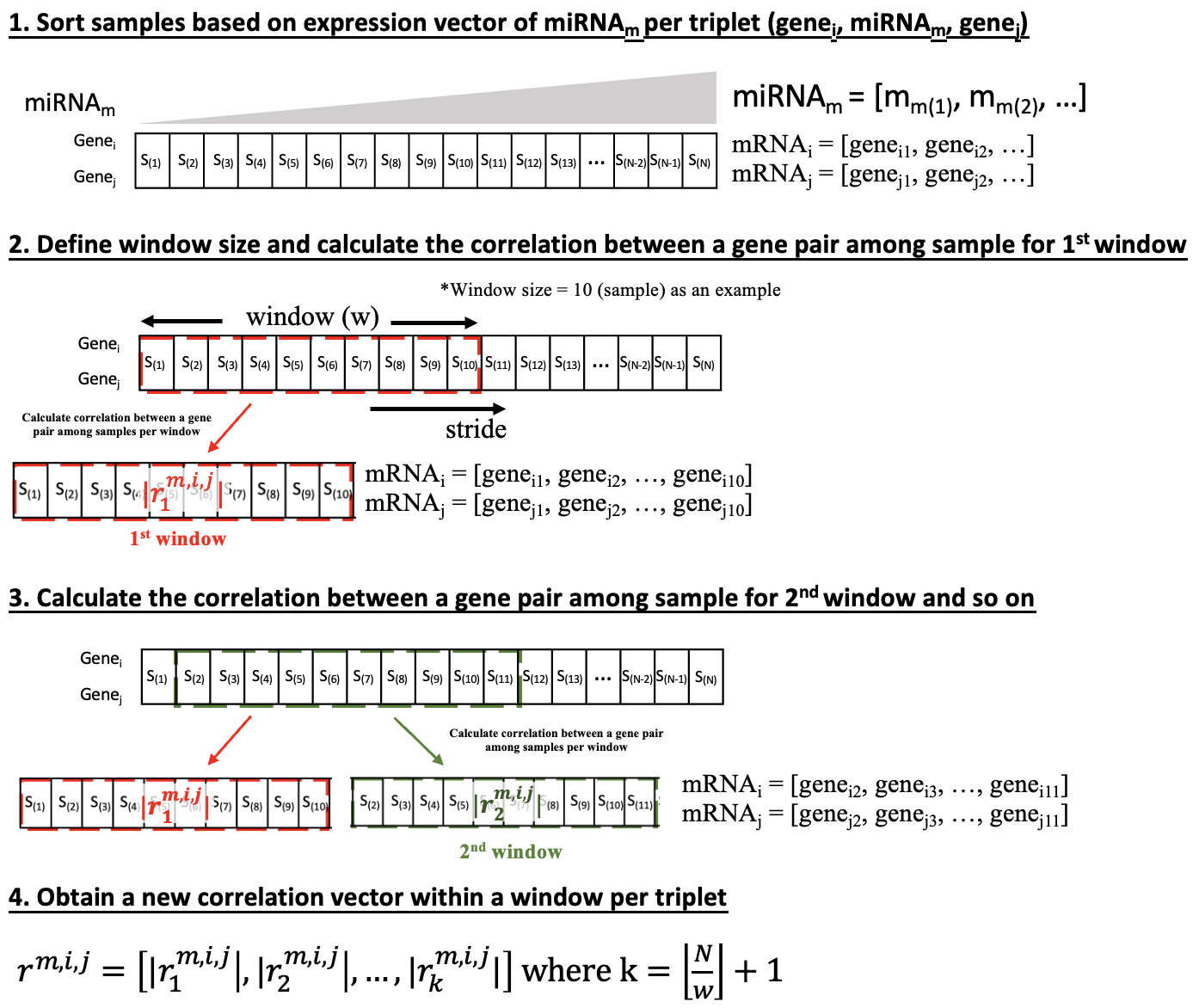

Supplement: S9 Fig — First, samples [S1, …SN] based on the expression vector of each miRNA per triplet (genei, miRNAm, genej) are sorted. Second, the window size is defined, and the correlation between gene pairs among samples for the first window is calculated. Next, the correlation between gene pairs among samples for the second window is calculated, and so on. Finally, a new correlation vector per triplet is created. (TIFF) [file pcbi.1010497.s009.tiff]

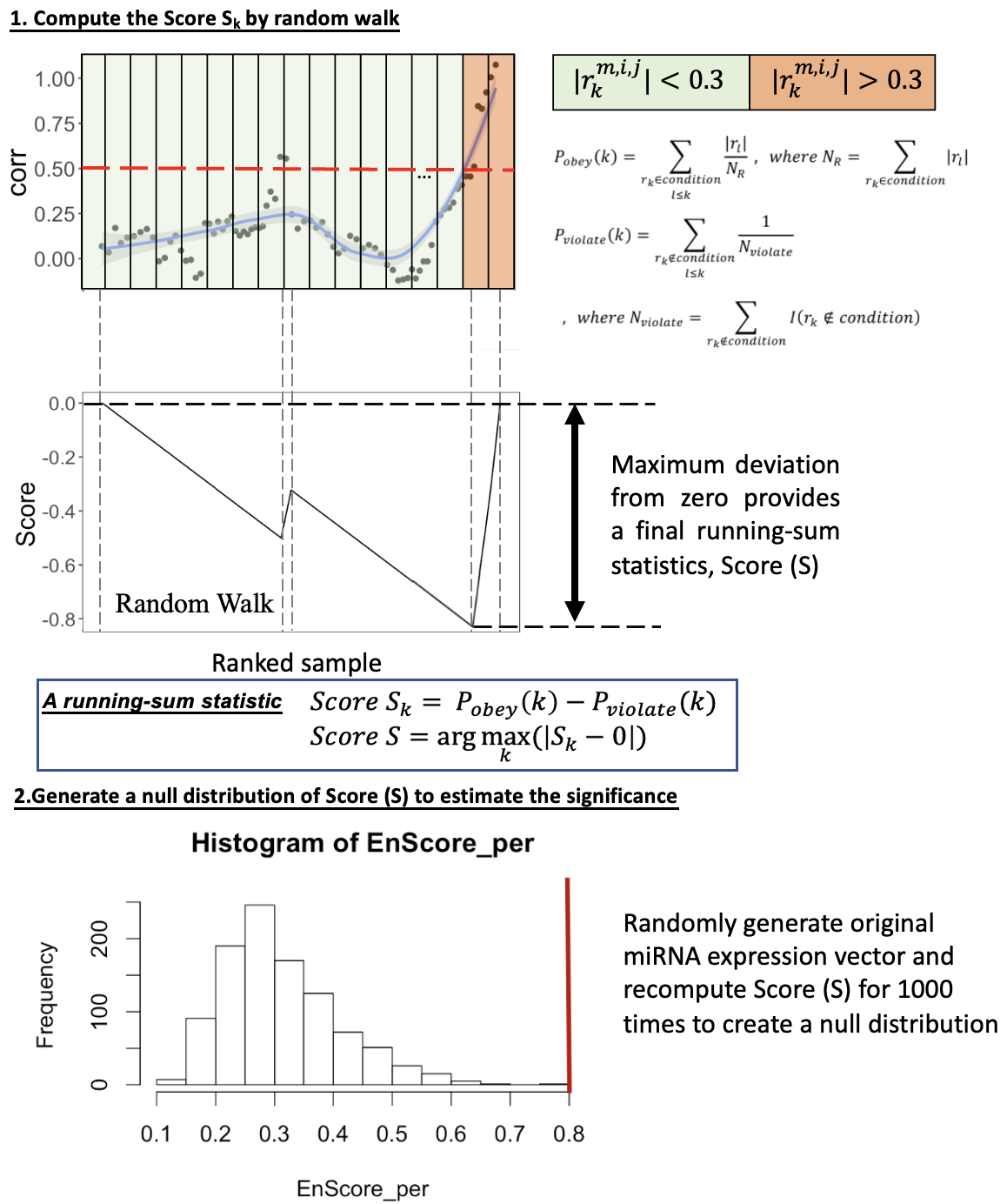

Supplement: S10 Fig — First, the k correlation values per triplet are ordered according to the average miRNA expression, miRNAk=∑n=0w−1mk+nm,i,j/w. The score (S) reflects the degree to which the correlation of a gene pair is overrepresented across all values (i.e., high/low/moderate miRNA expression) of the entire set of ranked miRNA expression levels. It is calculated by walking down the ranked value, increasing the score when encountering a specific miRNA expression value with the correlation of a paired gene over 0.3. If the correlation is less than 0.3 (threshold), the score will be negative. The magnitude of the score depends on the number of samples supported by such correlation values within a window. Subsequently, the permuted miRNA expression data are generated 1,000 times to create a null distribution of the S, and then the empirical P-value is calculated accordingly. (TIFF) [file pcbi.1010497.s010.tiff]

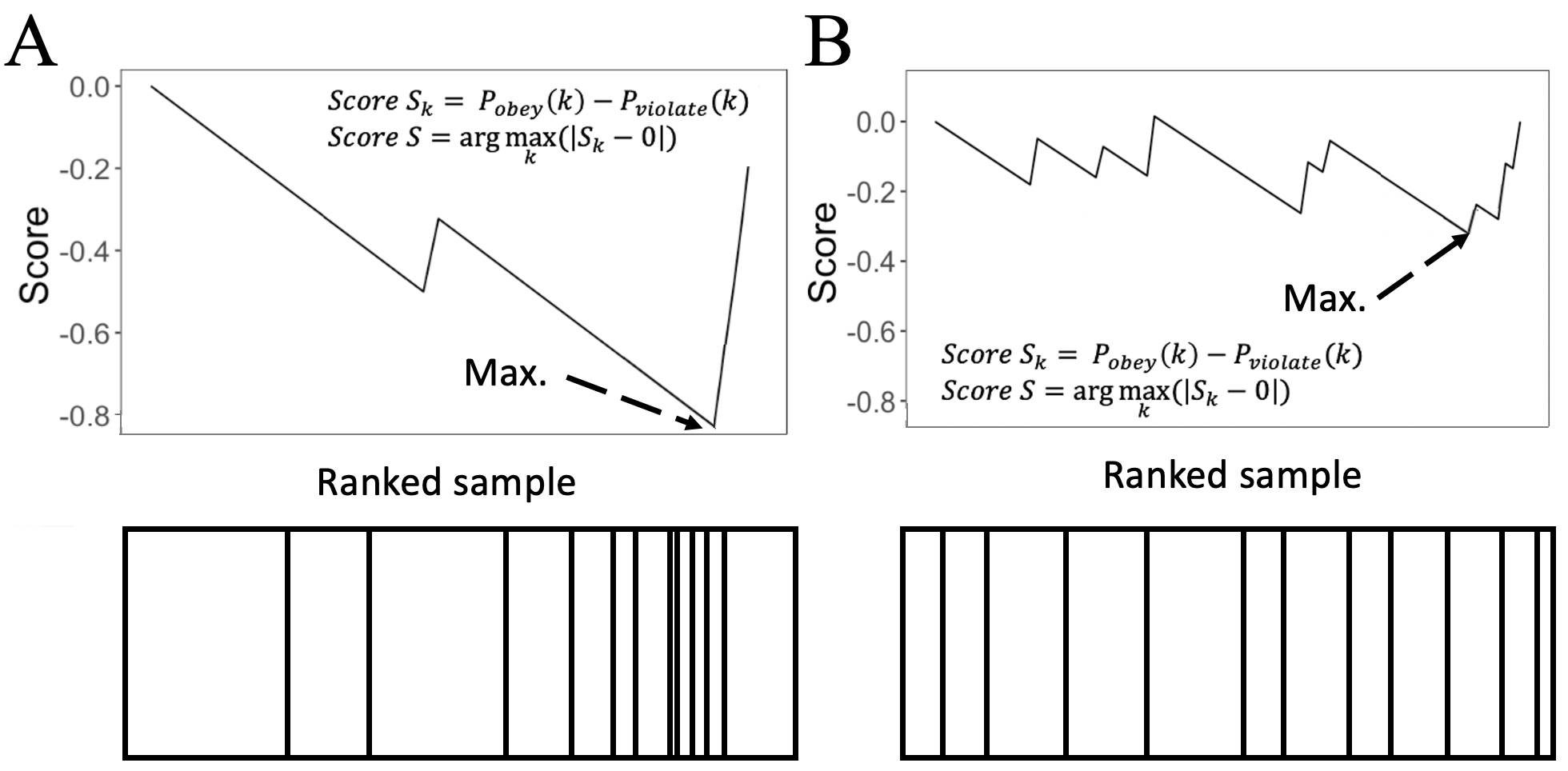

Supplement: S11 Fig — (A) Samples with the highest correlation between target genes are enriched at a higher miRNA expression value, supporting that these two target genes have a higher chance to compete with this miRNA. (B) These target gene pairs do not represent a biologically relevant correlation with the competition of this miRNA, suggesting that no ceRNA event occurs in this triplet. (TIFF) [file pcbi.1010497.s011.tiff]

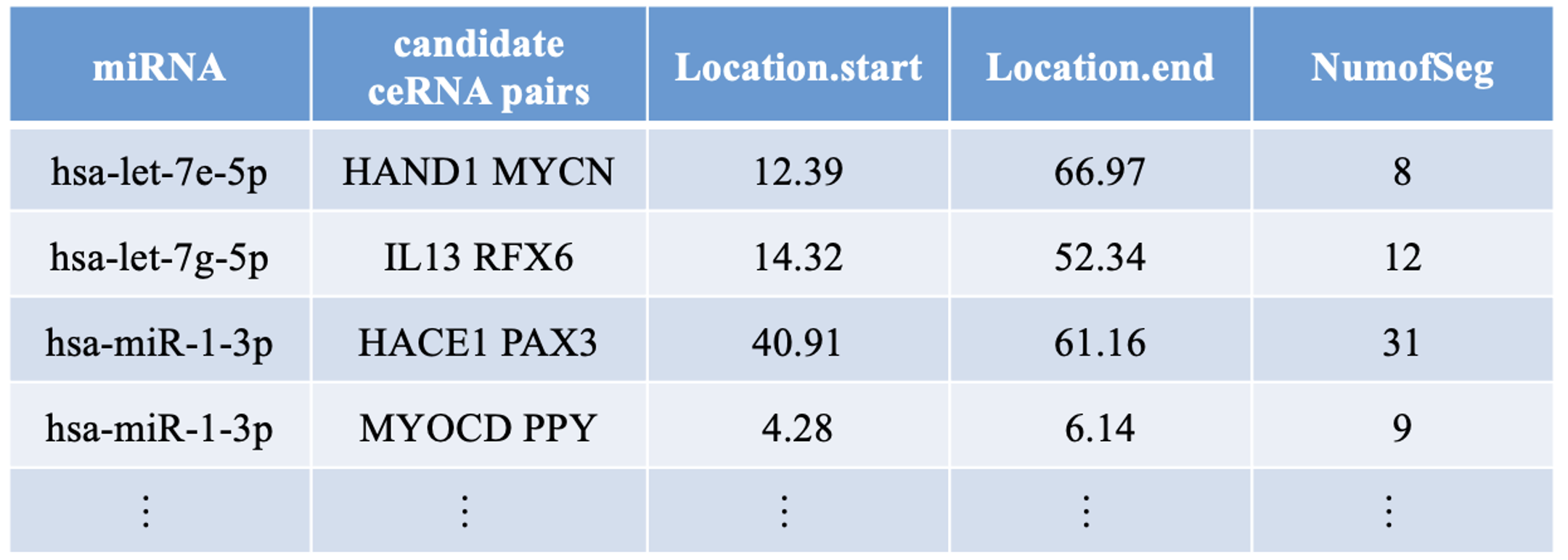

Supplement: S12 Fig — Five columns are involved: miRNA name, candidate ceRNA pairs, the start and end of each miRNA expression interval, and the number of segments. (TIFF) [file pcbi.1010497.s012.tiff]

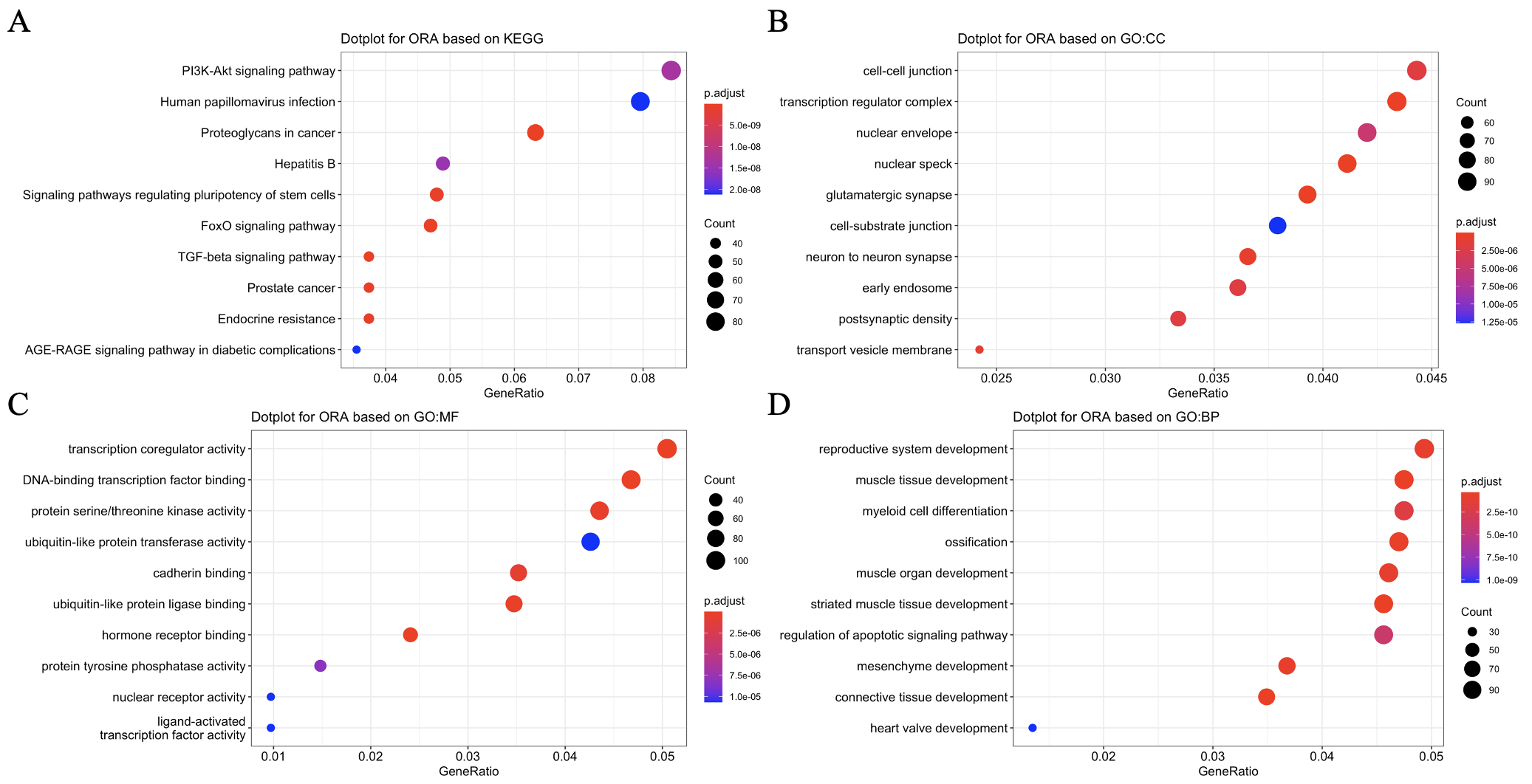

Supplement: S13 Fig — (A) Based on the KEGG database. (B) Based on the ‘cellular component’ (CC) categories in the gene ontology (GO) database. (C) Based on the ‘molecular function’ (MF) categories in the GO database. (D) Based on the ‘biological process’ (BP) categories in the GO database. (TIFF) [file pcbi.1010497.s013.tiff]

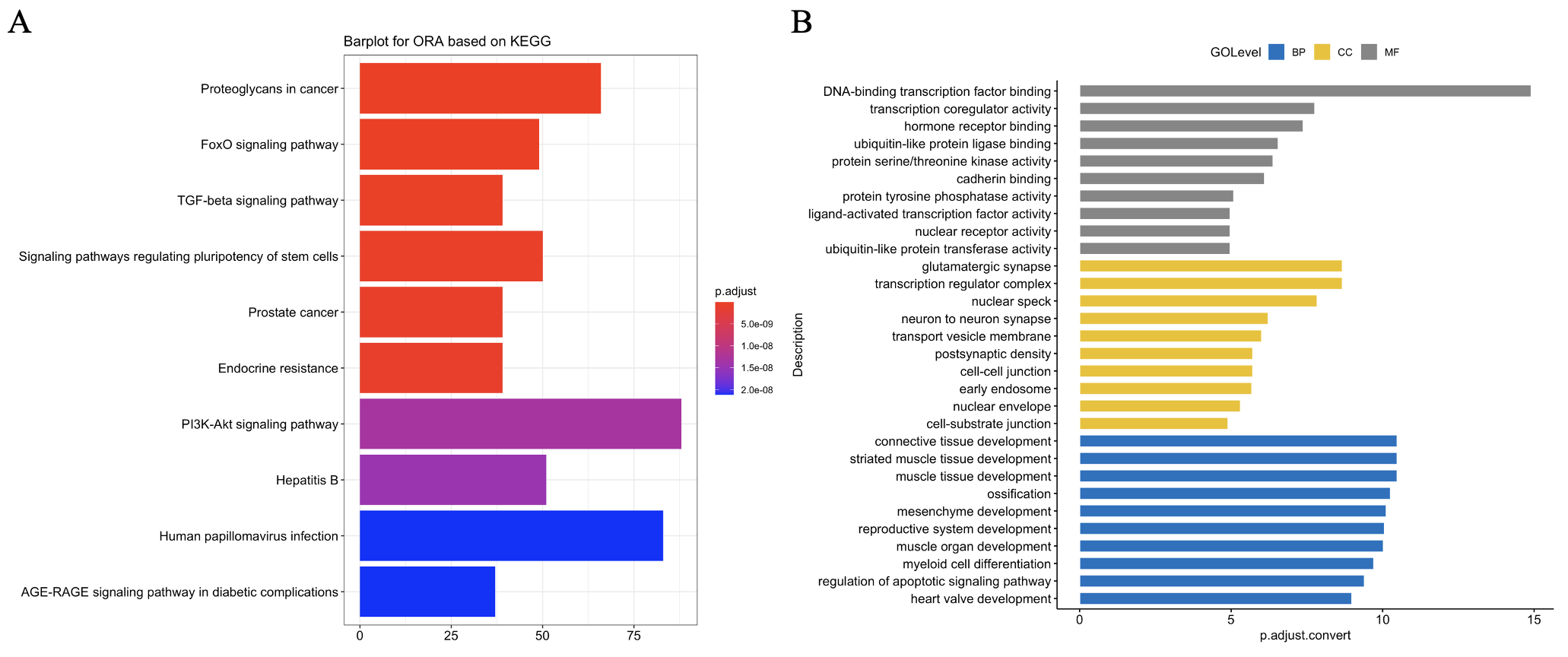

Supplement: S14 Fig — (A) Based on the KEGG database. (B) Based on the gene ontology (GO) database and stratified by three GO levels: cellular component (CC), molecular function (MF), and biological process (BP). (TIFF) [file pcbi.1010497.s014.tiff]

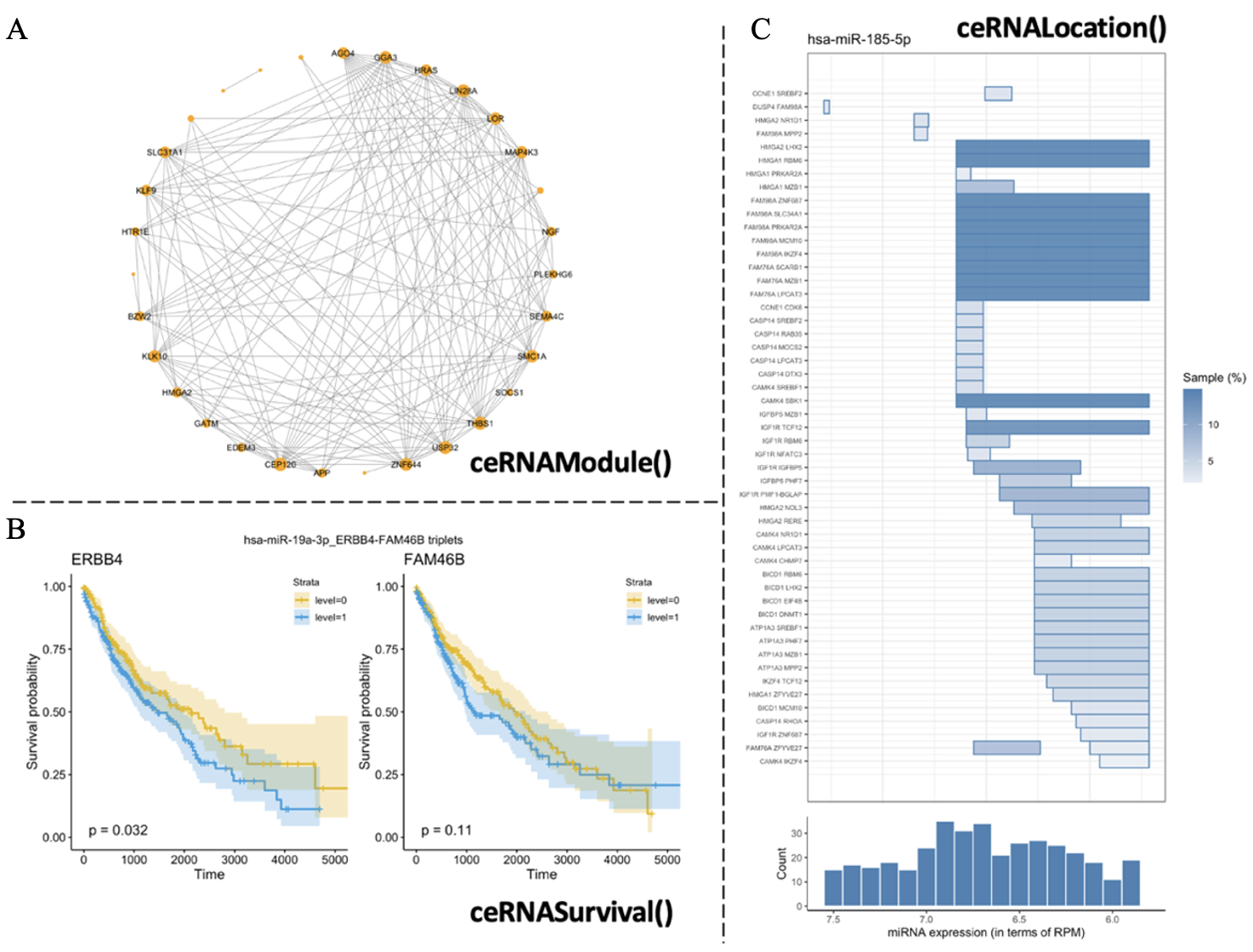

Supplement: S15 Fig — (A) Network analysis among candidate ceRNAs for a specific miRNA. (B) Survival analysis for a candidate ceRNA pair targeted by a specific miRNA. (C) A mix of a box plot and bar plot represents the proportion of all candidate ceRNA pairs targeted by a specific miRNA at their corresponding miRNA expression range. (TIFF) [file pcbi.1010497.s015.tiff]

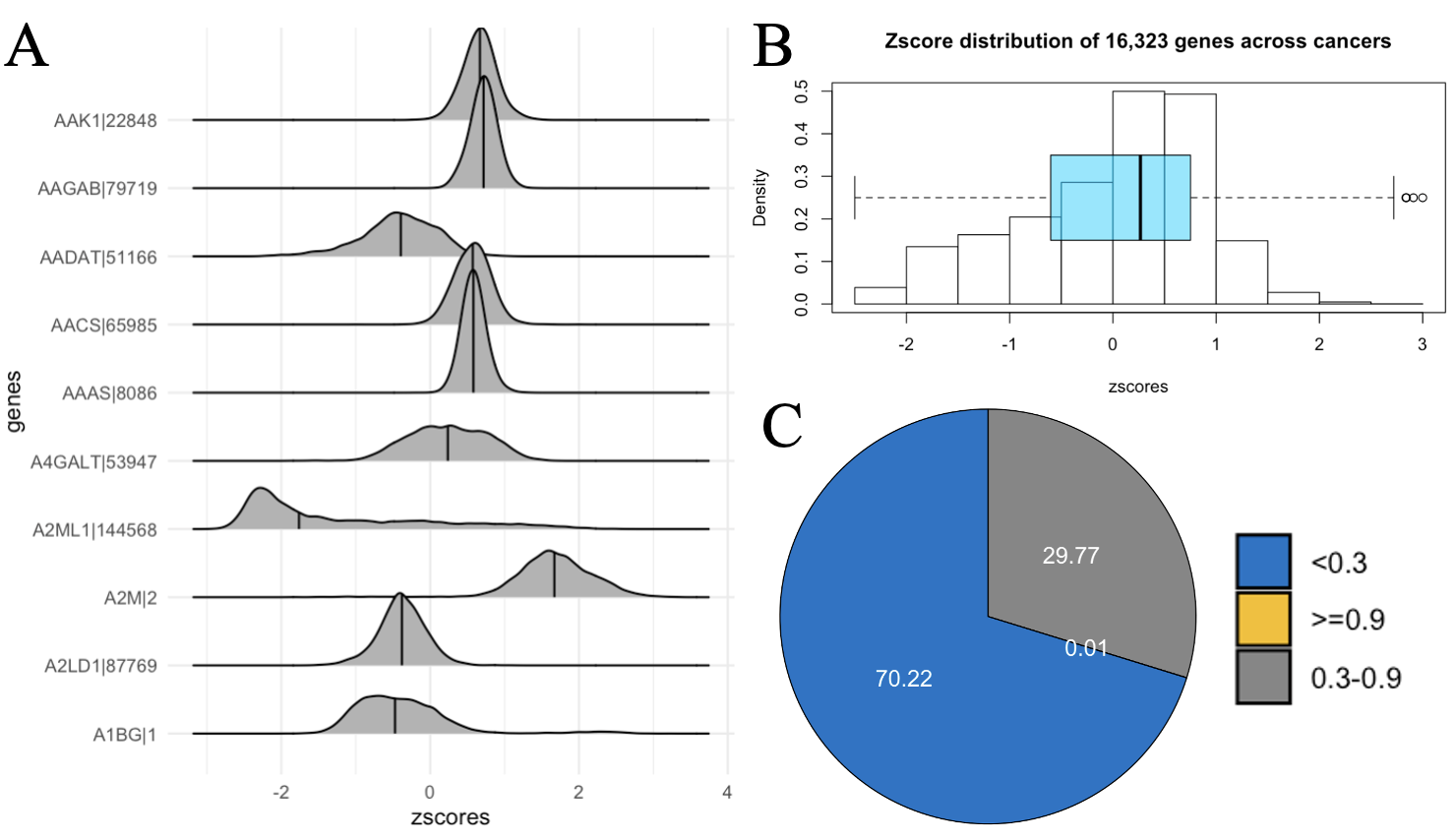

Supplement: S16 Fig — (A) The density plots represent the sample distribution of observed z-scores of 10 genes. (B) The density plot and boxplot represent the sample distribution of observed z-scores of 16,323 genes. (C) The pie chart shows the proportion of the Pearson correlation values of 16,323 genes. All the expression values are log-transformed and z-transformed across cancers. The median expression values of samples across cancers are used for summary statistics. (TIFF) [file pcbi.1010497.s016.tiff]
