# Supplementary material for: ceRNAR: An R package for identification and analysis of ceRNA-miRNA triplets
Source: PLoS Comput Biol. 2022 Sep 9;18(9):e1010497. doi: 10.1371/journal.pcbi.1010497 (PMC9491567; doi:10.1371/journal.pcbi.1010497)
Supplement: S15 Table — (DOCX) [file pcbi.1010497.s031.docx]

**S15 Table. Top bridging miRNAs and their corresponding hub gene among ceRNAs in the two TCGA datasets.**

| **TCGA dataset** | **miRNA** | **Bridging ceRNA triplets** | **Gene** | **ceRNA triplets** |
| --- | --- | --- | --- | --- |
| **TCGA-LUAD** | hsa-miR-19a-3p | 2396 | *DSEL* | 72 |
|  | hsa-miR-200b-3p | 1908 | *PDZD2* | 70 |
|  | hsa-miR-141-3p | 1778 | *ASTN1* | 64 |
|  | hsa-miR-27a-3p | 1514 | *SETD5* | 53 |
|  | hsa-miR-29a-3p | 1412 | *MEGF6* | 55 |
|  | hsa-miR-135a-5p | 569 | *PSIP1* | 35 |
|  | hsa-miR-23a-3p | 551 | *B4GAT1* | 33 |
|  | hsa-miR-199a-3p | 532 | *CHAF1B* | 37 |
|  | hsa-miR-30e-5p | 526 | *BMI1* | 32 |
|  | hsa-miR-125b-5p | 524 | *MAP3K11* | 32 |
|  | hsa-miR-183-5p* | 499 | *SMAD4* | 35 |
|  | hsa-miR-20a-5p | 491 | *IRF2* | 33 |
|  | hsa-miR-130a-3p | 490 | *TMEM159* | 37 |
|  | hsa-miR-153-3p | 436 | *CLMP* | 34 |
|  | hsa-miR-218-5p | 386 | *PKP4* | 28 |
|  | hsa-miR-133a-3p* | 356 | *PPY* | 34 |
|  | hsa-miR-142-5p |  | *TP53INP1* | 28 |
|  | hsa-miR-29b-3p | 348 | *PDGFRA* | 30 |
|  | hsa-miR-19b-3p | 330 | *NCOA3* | 25 |
|  | hsa-miR-138-5p | 324 | *TPM4* | 28 |
| **TCGA-LUSC** | hsa-miR-148a-3p | 1049 | *ATP11A* | 45 |
|  | hsa-miR-183-5p* | 711 | *CCDC121* | 35 |
|  | hsa-miR-133a-3p* | 382 | *ASH1L* | 30 |
|  | hsa-miR-30a-5p | 380 | *ABL1* | 28 |
|  | hsa-miR-205-5p | 368 | *ACSL1* | 25 |
|  | hsa-miR-23b-3p | 348 | *ADGRG2* | 22 |
|  | hsa-miR-302c-3p | 324 | *CRTC2* | 24 |
|  | hsa-miR-17-5p | 304 | *CCND2* | 25 |
|  | hsa-miR-140-3p | 300 | *BRD3* | 27 |
|  | hsa-miR-142-3p | 288 | *C9orf72* | 26 |
|  | hsa-miR-142-5p* | 270 | *ALS2* | 19 |
|  | hsa-miR-128-3p | 245 | *BAX* | 18 |
|  | hsa-miR-338-3p | 225 | *ETS1* | 15 |
|  | hsa-miR-217 | 211 | *BORCS5* | 21 |
|  | hsa-miR-301a-3p | 209 | *ASXL2* | 17 |
|  | hsa-miR-29c-3p | 189 | *AKT2* | 15 |
|  | hsa-let-7d-5p | 171 | *CPEB1* | 20 |
|  | hsa-let-7a-5p | 166 | *CEP120* | 20 |
|  | hsa-miR-30d-5p | 164 | *BCL9* | 20 |
|  | hsa-miR-10a-5p | 146 | *FUT1* | 13 |

* These gene were found in both datasets.
