# Supplementary material for: ceRNAR: An R package for identification and analysis of ceRNA-miRNA triplets
Source: PLoS Comput Biol. 2022 Sep 9;18(9):e1010497. doi: 10.1371/journal.pcbi.1010497 (PMC9491567; doi:10.1371/journal.pcbi.1010497)
Supplement: S16 Table — (DOCX) [file pcbi.1010497.s032.docx]

**S16 Table. Hub genes among ceRNA triplets in the two TCGA datasets.**

| **TCGA dataset** | **Gene** | **ceRNA triplets** |
| --- | --- | --- |
| **TCGA-LUAD** | *GALNT15* | 286 |
|  | *TP53INP1* | 265 |
|  | *PDGFRA* | 254 |
|  | *RNF38* | 227 |
|  | *TBL1XR1* | 210 |
|  | *FOXP1* | 195 |
|  | *TRAPPC8* | 193 |
|  | *MAP4K3** | 190 |
|  | *CFL2* | 189 |
|  | *CPEB3* | 189 |
| **TCGA-LUSC** | *KLK10* | 69 |
|  | *BZW2* | 68 |
|  | *HMGA2* | 65 |
|  | *KLF9* | 60 |
|  | *ATP2A2* | 58 |
|  | *MAP4K3** | 56 |
|  | *ZNF644* | 55 |
|  | *GGA3* | 53 |
|  | *PLEKHG6* | 52 |
|  | *TARBP2* | 49 |

* These gene were found in both datasets.
