# Supplementary material for: ceRNAR: An R package for identification and analysis of ceRNA-miRNA triplets
Source: PLoS Comput Biol. 2022 Sep 9;18(9):e1010497. doi: 10.1371/journal.pcbi.1010497 (PMC9491567; doi:10.1371/journal.pcbi.1010497)
Supplement: S17 Table — (DOCX) [file pcbi.1010497.s033.docx]

**S17 Table. Comparisons with the state-of-the-art tools.**

| **Features** | **ceRNAR** | **SPONGE** | **GDCRNATools** | **CERNIA** | **JAMI** | **Cupid** |
| --- | --- | --- | --- | --- | --- | --- |
| **miRNA-targets data sources** | miRTarBase, miRecords, DIANA-micro T-CDS, EIMMO, miRDB, miRanda, PITA, RNA22, TargetScan | User-provided e.g. TargetScan, miRcode, LncBase, mirTarBase | StarBase, miRcode,  miRTarBase, spongeScan | User-provided e.g. miRTarBase, starBase,  miRecords, miRanda, DT-Hybrid | miRecords, TarBase,  TRANSFAC, miRnada, PITA, Targetcan | miRecords, TarBase,  TRANSFAC, miRnada, PITA, Targetcan |
| **TCGA expression profile** | Yes | Yes | Yes | Yes | Yes | Yes |
| **ceRNA classes** | mRNA | mRNA, lncRNA | mRNA, lncRNA | mRNA,lncRNA, pseudogene | mRNA | mRNA |
| **ceRNA prediction algorithm** | Pearson correlation +  Data augmentation + Running sum statistics | Sensitivity correlation for  more than one miRNA | Hypergeometric test +  Pearson correlation +  Regulation similarity | MRE-based +  Gene-expression correlation +  SVM classification | Non-parameter + Multi-threading version of Cupid | MRE-based +  conditional mutual information |
| **P-value on ceRNA interaction score** | Yes | Yes | No | No | Yes | Yes |
| **Implementation** | R language | R language | R language | R language | Java, R language | Matlab |
| **Genome-wide screening** | Yes | Yes | Yes | Yes | Yes | Yes |
| **Reference** | N/A | M. List et al. 2019 | R. Li et al. 2018 | D. S. Sardina et al. 2017 | A. Hornakova et al. 2018 | H. Chiu et al. 2014 |
