# Supplementary material for: ceRNAR: An R package for identification and analysis of ceRNA-miRNA triplets
Source: PLoS Comput Biol. 2022 Sep 9;18(9):e1010497. doi: 10.1371/journal.pcbi.1010497 (PMC9491567; doi:10.1371/journal.pcbi.1010497)
Supplement: Attachment — Submitted filename: 5.rebuttal_letter_cernar_plos edited.docx [file pcbi.1010497.s034.docx]

The experts that have reviewed your manuscript has raised a number of convergent concerns, including major ones about the lack of a comparison with existing methods and the insufficient description of the algorithmic procedure. You are invited to review the manuscript taking all revewer's recommendation into account, including a systematic comparison with established method to demonstrate in objective terms the additional insight provided by ceRNAR.

Upon resubmission, your manuscript will be returned to the three reviewers.
C. Micheletti
 We appreciate the comments from the editor and the 3 reviewers. These two main concerns have been further addressed in this revised version. We have emphasized the rationale behind our approach in the Materials and Methods section and provided more graphic illustrations for the readers to have a better intuition of our methods (S8-11 Figs and associated text). Also, we have compared our methods with other tools in terms of the features, performance, and running time (Fig 3, S4 Fig and S17 Table and associated text). In addition, we’ve sent the manuscript for English editing by a native speaker. The certificate has been uploaded into the system for your reference.

(line 397-399) ‘The purpose of this function is to identify ceRNA-miRNA triplets based on the Pearson correlation coefficient through the sliding window approach (S9 Fig) and a running sum statistic for such values by the random walk approach (S10 Fig).’

(line 305-408) ‘Technically, the correlation value between two genes is usually calculated using the gene expression values of all samples. By using a sliding window approach [45], we can artificially create different varieties of a real dataset to increase its size, which is a sort of data augmentation technique [46].’

(line 422-431) ‘S is then defined as the maximum distance from zero of the substations of P_obey_(k) from P_violate_(k). If the samples with similar correlation values are not enriched at a particular miRNA expression interval, it means those gene pairs are not biologically relevant to compete with miRNA in a miRNA-gene triplet; that is, there is no ceRNA event observed in this triplet (S11 Fig). Finally, we accessed the significance of an observed S by comparing it with the theoretical S computed by randomly permutating the expression of the candidate miRNA_m_ 1,000 times to provide assessment of significance. When an entire sample of potential triplets is evaluated, these p-values will be adjusted by the false discovery rate, which provides the estimated probability of whether a triplet within an entire set of potential triplets is a false positive finding or not.’

(line 155-171) ‘We have compared our tool with other state-of-the-art tools, including SPONGE, JAMI, GDCRNATools, and CERNIA, in terms of their performances using synthetic data and their running time using real data. Fig 3A and S12 Table illustrate that ceRNAR workflow generally outperformed the other tools in terms of sensitivity and PPV in all scenarios when the window size is set to 10 and the correlation cutoff is set to 0.7. It can be mainly observed when the correlation values among correlated genes are relatively high. However, all of the tools could identify valid ceRNA triplets without reporting high false-positive results except JAMI (Fig 3B and S12 Table). GDCRNATools generally had a high sensitivity compared to the other tools, and a lower specificity than ceRNAR, suggesting that it is good for catching ceRNAs but comes with a relatively high rate of false positives. Regarding running time, we used different sample numbers (250, 500, and 100) on a small subset of the pan-cancer dataset with 15 genes which form 105 triplets; we also used different triplet numbers (105, 1,225, and 4,950) on a small subset of the pan-cancer dataset with 250 samples (S4 Fig and S13 Table). Although the ceRNA algorithm was not fast with a large sample size and a large number of triplets compared with SPONGE, GDCRNATools and CERNIA, it was slightly faster than JAMI.’

(line 238-266) Recently, more and more tools have been developed to identify potential ceRNA events. It is important to systematically evaluate the ceRNAR package in comparison with the five other published ceRNA prediction tools—SPONGE [20], CERNIA [21], GDCRNATools [22], JAMI [23], and CUPID [24]—that are expression-based (rather than sequence-based, i.e., spongeScan [25]). We have compared them in terms of several features, such as miRNA-target data sources, study design, ceRNA classes, ceRNA prediction algorithm, and language for implementation (S17 Table). Noting that JAMI is the multi-threading version of CUPID, we decided to keep only JAMI for further analyses. Thus, we used four algorithms, including SPONGE, CERNIA, GDCRNATools, and JAMI, for the comparisons. Notably, the four algorithms and our ceRNAR all used a similar strategy, which is utilizing miRNA-target data sources to identify potential miRNA-gene/lncRNA pairs from other databases. All five of these algorithms are implemented in R and consider the correlations between miRNA-mediated genes/lncRNA. It is worth mentioning that CERNIA and JAMI consider both MRE- and expression-based data, whereas SPONGE, GDCRNATools and ceRNAR only analyze genome-wide expression data. Although these five algorithms are all correlation-based, some differences still exist. For examples, JAMI is based on conditional mutual information, which is particularly useful to capture non-linear associations by estimating the effect of a miRNA on its target pairs through a permutation test [26], while GDCRNATools is based on sensitivity correlation computation through effectively estimating covariate matrices and also considering the impact of a miRNA on its target pairs. SPONGE also uses sensitivity correlation to quantify the impact of a miRNA on its target pairs (i.e., linear partial correlation), but further applies a null model-based p-value computation to estimate potential ceRNA pairs. Since several studies [18, 27, 28] indicate that ceRNA triplets may be observed in a specific range of miRNA expression, such an approach can help to focus on the true positive region with high signal-to-noise ratio instead of missing the ceRNA triplets due to signal dilution by global noise. Our ceRNAR algorithm showed highest sensitivity in identify potential ceRNA triplets (Fig 3).

**S8 Fig. The basic concept of our idea is illustrated graphically.** (A) Lists of curated miRNAs and their targets are verified from nine miRNA target databases based on the AnamiR package and selected by being either experimentally validated or present in over half of the prediction databases. (B) Our algorithm iteratively evaluates whether each mRNA pair is a potential ceRNA event in each miRNA-target list.

**S9 Fig. A graphic overview illustrates sorting and correlation calculation in the *ceRNApairFiltering* method.** First, samples based on the expression vector of each miRNA per triplet (genei, miRNAm, genej) are sorted. Second, the window size is defined, and the correlation between gene pairs among samples for the first window is calculated. Next, the correlation between gene pairs among samples for the second window is calculated, and so on. Finally, a new correlation vector per triplet is created.

**S10 Fig. A graphic overview illustrates the random walk and permutation test in the *ceRNApairFiltering* method.** First, the k correlation values per triplet are ordered according to the average miRNA expression, ${miRNA}_{k}={\sum_{n=0}^{w-1} m_{k+n}^{m,i,j}}/w$. The score (S) reflects the degree to which the correlation of a gene pair is overrepresented across all values (i.e., high/low/moderate miRNA expression) of the entire set of ranked miRNA expression levels. It is calculated by walking down the ranked value, increasing the score when encountering a specific miRNA expression value with the correlation of a paired gene over 0.3. If the correlation is less than 0.3 (threshold), the score will be negative. The magnitude of the score depends on the number of samples supported by such correlation values within a window. Subsequently, the permuted miRNA expression data are generated 1,000 times to create a null distribution of the S, and then the empirical P-value is calculated accordingly.

**S11 Fig. The meanings of score S are based on the values of P_obey_ and P_violate_.** (A) Samples with the highest correlation between target genes are enriched at a higher miRNA expression value, supporting that these two target genes have a higher chance to compete with this miRNA. (B) These target gene pairs do not represent a biologically relevant correlation with the competition of this miRNA, suggesting that no ceRNA event occurs in this triplet.

**Fig 3. Performance comparisons between ceRNIA, JAMI, SPONGE, GDCRNATools and ceRNAR packages using synthetic data that mimic the real world case.** (A) Sensitivity and PPV of various tools at different correlation levels for 100 samples of 105 pairs of target genes under four scenarios. (B) Specificity and NPV of various tools at different correlation levels for 100 sample of 105 pairs of target genes under null scenarios.

**S4 Fig. Run time comparisons between published tools and our packages.** (A) Run time for different sample numbers on a fixed set of genes using real data. (B) Run time for different numbers of genes (i.e., different numbers of triplets) on a fixed number of samples using real data.

**S17 Table. Comparisons with the state-of-the-art tools.**

| **Features** | **ceRNAR** | **SPONGE** | **GDCRNATools** | **CERNIA** | **JAMI** | **Cupid** |
| --- | --- | --- | --- | --- | --- | --- |
| **miRNA-targets data sources** | miRTarBase, miRecords, DIANA-micro T-CDS, EIMMO, miRDB, miRanda, PITA, RNA22, TargetScan | TargetScan v6.0, miRcode | StarBase v2.0, miRcode,  spongScan | miRTarBase, starBase,  miRecords, miRanda, DT-Hybrid | miRecords, TarBase,  TRANSFAC | miRecords, TarBase,  TRANSFAC |
| **TCGA expression profile** | Yes | Yes | Yes | Yes | Yes | Yes |
| **Study design** | Case-only | Case-only | Case-only | Case-only | Case-only | Case-only |
| **ceRNA classes** | miRNA-mRNA | miRNA-mRNA/lncRNA | miRNA-lncRNA | miRNA-mRNA/ | miRNA-mRNA | miRNA-mRNA |
| **ceRNA prediction algorithm** | Pearson correlation +  Data augment + Running sum statistics | Sensitivity correlation for  more than one miRNA | Hypergeometric test +  Pearson correlation +  Regulation similarity | MRE-based +  gene-expression correlation +  SVM classification | Non-parameter + multi-threading version of Cupid | MRE-based +  conditional mutual information |
| **P-value on ceRNA interaction score** | Yes | Yes | No | No | Yes | Yes |
| **Implementation** | R language | R language | R language | R language | Java, R language | Matlab |
| **Genome-wide screening** | Yes | Yes | Yes | Yes | Yes | Yes |
| **Reference** | N/A | M. List et al. 2019 | R. Li et al. 2018 | D. S. Sardina et al. 2017 | A. Hornakova et al. 2018 | H. Chiu et al. 2014 |

Reviewer's Responses to Questions

**Comments to the Authors:
Please note here if the review is uploaded as an attachment.**

Reviewer #1: Authors present ceRNAR, an algorithm, written in R, that is able to predict ceRNA-miRNA triplets. Competitive endogenous RNA (ceRNA) are RNA molecules involved in the gene regulation related to several biological and pathological processes. In particular, they form with a miRNA a small interaction network called ceRNA-miRNA triplets, composed of two RNAs and one miRNA. ceRNAR is based on a correlation approach, and moreover it provides some software modules in order to make downstream analysis over the predicted ceRNA networks, including gene enrichment and survival analysis. Experimental tests were carried out using both simultated and real case datasets. The results, in terms of accuracy, sensitivity and specificity, on simulated studies are good.
The paper is clear and well written. English is good. In my opinion, the experimental part of the paper needs to be improved.

Major remarks:

- It is not clear what is the ground truth in your simulation studies. You should better explain that. Moreover, why did you not consider repository of experimentally validate ceRNA interactions collected in databases like miRsponge ([http://bio-bigdata.hrbmu.edu.cn/miRSponge/](about:blank)) in order to validate ceRNAR? The simulation study is not enough.
 We appreciate the suggestion, and we have added the validation analyses using those experimentally validated ceRNA interactions collected in miRsponge. However, there are only 158 experimentally validated ceRNA triplets in miRsponge, which is too few to be analyzed. Hence, we used experimentally validated miRNA-target pairs (total pairs: 6,497) collected in miRsponge to do the analysis. An R function *ceRNAValidate()* was further created to link our findings to the experimentally validated results from the miRsponge database. We also used graphic outputs to summarize those experimentally validated results supporting the potential ceRNA events identified by the ceRNAR algorithm (S7 Fig in the text). As shown in S7 Fig, around 1% of experimentally validated ceRNA triplets were identified in LUAD and LUSC. The low proportion may be attributed to the fact that only 158 ceRNA triplets were analyzed and also that those ceRNA triplets may not be expressed in lung tissues. Furthermore, approximately 13-14% of ceRNA triplets with at least one experimentally validated miRNA-target pair were identified by using the ceRNAR algorithm. A Chi-square test was performed to examine whether the findings from the ceRNAR package were significantly enriched in the database, and the results showed that the P-values obtained from the LUAD and LUSC datasets were both less than 2.2e-16, suggesting our ceRNAR package can successfully identify previously reported ceRNA triplets.

(line 497-499) ‘We also implemented an integrated function (*ceRNAIntegrate*) to combine our results with other state-of-the-art tools, such as SPONGE [20] and JAMI [23], and a validation function (*ceRNAValidate*) based on the miRSponge database.’

(line 200-212) ‘In addition, we compared our findings against experimentally validated miRNA-gene pairs in the miRSponge database to endorse the potential ceRNAs identified by ceRNAR. As shown in S7 Figure, around 1% (ceRNA pairs that can be validated among total ceRNA founded based on ceRNAR algorithm) of experimentally validated ceRNA triplets were identified in LUAD and LUSC. The low proportion may be attributed to the fact that only 158 ceRNA triplets were analyzed and that those ceRNA triplets may not be expressed in lung tissues. Furthermore, approximately 13-14% of ceRNA triplets with at least one experimentally validated miRNA-target interaction were identified by using the ceRNAR algorithm. A Chi-square test was performed to examine whether the findings from the ceRNAR package were significantly enriched in the TCGA data, and the results showed that the P-values obtained from the LUAD and LUSC datasets were both less than 2.2e-16, suggesting our ceRNAR package can successfully identify previously reported ceRNA triplets.’

**Fig 4. An overview of our ceRNAR package.** This package includes three main parts. First, “Input data” has three functions: *ceRNAputativePairs()* for extracting curated miRNA-target lists, *ceRNATCGA()* for retrieving TCGA data, and *ceRNAcustomize()* for importing customized data. Second, “Identification of miRNA-ceRNA triplets” involves *ceRNApairFiltering()* and *SegmentClusteringPlusPeakMerging()* and is wrapped into a *ceRNAmethod()* function. Lastly, “Downstream analyses” contains six functions for different tasks, that is, *ceRNAFunction(), ceRNAModule(), ceRNASurvival(), ceRNALocation(), ceRNAValidate()* and *ceRNAIntegrate()*.

**S7 Fig. Experimental validation of real case study based on miRSponge database.** (A) TCGA-LUAD (The Cancer Genome Atlas Lung Adenocarcinoma). (B) TCGA-LUSC (The Cancer Genome Atlas Lung Squamous Cell Carcinoma).

- Authors did not compare ceRNAR with other existing ceRNA predictors. Although in the Introduction they cited several similar tools, no comparison has been done. For that reason, authors shoud add comparison with tools such as SPONGE [1], CERNIA [2], GDCRNATools [3], spongeScan ([http://spongescan.rc.ufl.edu/](about:blank)), and specify the added value ceRNAR can provide with regards to web serivices such as miRTissue_ce [4] and Encori ([https://starbase.sysu.edu.cn/](about:blank)).
 We sincerely appreciated the suggestion and have added “Comparison with other tools” in the Results section. We excluded the spongeScan algorithm because it requires sequence-based input data rather than expression profiles. In that case, the comparisons would be not fair due to significantly different starting points of input data. We have systematically evaluated five other published ceRNA prediction tools, including SPONGE, CERNIA, GDCRNATools, and CUPID / JAMI, in terms of many different features, including miRNA-target data sources, suitable study design, prediction algorithm, and implementation language (S17 Table). Note that JAMI is the multi-threading version of CUPID and thus we decided to exclude CUPID from further analyses; we focused on the remaining four algorithms, SPONGE, CERNIA, GDCRNATools, and JAMI, for the comparisons. Notably, these four algorithms and our ceRNAR all used a similar strategy, which is utilizing miRNA-target data sources to identify potential miRNA-gene/lncRNA pairs from other databases. All five algorithms are implemented in R and consider the correlations between miRNA-mediated genes/lncRNA. It is worth mentioning that CERNIA and JAMI consider both MRE- and expression-based data, whereas SPONGE, GDCRNATools and our ceRNAR only analyze genome-wide expression data. Although these five algorithms are all correlation-based, some differences still exist. For example, JAMI is based on conditional mutual information, which is particularly useful to capture non-linear associations by estimating the effect of a miRNA on its target pairs through a permutation test, while GDCRNATools is based on sensitivity correlation computation and effectively estimates covariate matrices and also considers the impact of miRNA on its target pairs. SPONGE also uses sensitivity correlation to quantify the impact of a miRNA on its target pairs (i.e., linear partial correlation), but it further applies a null model-based p-value computation to estimate potential ceRNA pairs. Compared with these four algorithms, the most important advantage of ceRNAR is that we considered a specific range of miRNA expression instead of the whole expression spectrum. Since several published studies [1-3] indicate that ceRNA triplets may be observed in a specific range of miRNA expression, such an approach can help to focus on the true positive region with high signal-to-noise instead of missing the ceRNA triplet due to dilution from global noise. Our ceRNAR algorithm showed the highest sensitivity in identifying potential ceRNA triplets (Fig 3 in the text). Fig 3A and S12 Table illustrate that ceRNAR’s workflow generally outperformed the other tools in terms of sensitivity and PPV in all scenarios when the window size was set to 10 and the correlation cutoff was set to 0.7. It can be mainly observed when the correlation values among genes are relatively high. However, all of the tools could identify valid ceRNA triplets without reporting high false-positive results except JAMI (Fig 3B and S12 Table). GDCRNATools generally had a higher sensitivity (except at higher correlation levels) but lower specificity than ceRNAR, suggesting that it is good for catching ceRNAs but comes with a relatively high rate of false positives. Regarding running time, we used different sample numbers (250, 500, and 100) on a small subset of the pan-cancer dataset with 15 genes, which form 105 triplets; we also used different triplet numbers (105, 1,225, and 4,950) on a small subset of the pan-cancer dataset with 250 samples (S4 Fig and S13 Table). Although the ceRNA algorithm cannot be computed fast for a large sample size and a large number of triplets compared with SPONGE, GDCRNATools and CERNIA, it was slightly faster than JAMI.

Regarding the two online servers, miRTissue_ce and Encori (i.e., starBase v2) are two web servers that integrate ceRNA integration data sources, ceRNA prediction algorithms, and even some data analyses and visualizations that can be easily accessed by the users. Fiannaca *et al.* 2020 have compared miRTissue_ce and Encori in terms of many features. Here, we further compared these web servers with our ceRNAR based on these features to see whether there is any add-on value that ceRNAR can provide. First, the interactions between miRNAs and target genes in ceRNAR are supported by nine databases, including two experimentally validated miRNA-target databases and seven computationally predicted miRNA-target databases. In contrast, Encori and miRTissue_ce are supported by 4 and 8 computationally predicted miRNA-target databases, respectively. Second, Encori uses a hypergeometric test to predict ceRNA, and miRTissue_ce integrates that method with a global test and SPONGE. However, one major disadvantage of the hypergeometric test is that the test requires a pre-defined p-value threshold to select significant genes. Such an approach is not suitable to be applied to the whole genome, because the hypergeometric test fails to consider the interactions among genes due to its independency assumption about genes. That is the reason why we present a novel rank-based algorithm considering the contribution of miRNA expression in a ceRNA event and extend the pair-wise correlation approach to identify ceRNA-miRNA triplets using whole genomic information. Lastly, these two web servers can predict many types of ceRNA, but ceRNAR only focuses on one ceRNA class (i.e., mRNA-miRNA).

[1] Xu J, Li Y, Lu J, Pan T, Ding N, Wang Z, et al. The mRNA related ceRNA–ceRNA landscape and significance across 20 major cancer types. Nucleic acids research. 2015;43(17):8169-82.

[2] Ala U, Karreth FA, Bosia C, Pagnani A, Taulli R, Léopold V, et al. Integrated transcriptional and competitive endogenous RNA networks are cross-regulated in permissive molecular environments. Proceedings of the National Academy of Sciences. 2013;110(18):7154-9.

[3] Bosson AD, Zamudio JR, Sharp PA. Endogenous miRNA and target concentrations determine susceptibility to potential ceRNA competition. Molecular cell. 2014;56(3):347-59.

(line 155-171) ‘We have compared our tool with other state-of-the-art tools, including SPONGE, JAMI, GDCRNATools, and CERNIA, in terms of their performances using synthetic data and their running time using real data. Fig 3A and S12 Table illustrate that ceRNAR workflow generally outperformed the other tools in terms of sensitivity and PPV in all scenarios when the window size is set to 10 and the correlation cutoff is set to 0.7. It can be mainly observed when the correlation values among correlated genes are relatively high. However, all of the tools could identify valid ceRNA triplets without reporting high false-positive results except JAMI (Fig 3B and S12 Table). GDCRNATools generally had a high sensitivity compared to the other tools, and a lower specificity than ceRNAR, suggesting that it is good for catching ceRNAs but comes with a relatively high rate of false positives. Regarding running time, we used different sample numbers (250, 500, and 100) on a small subset of the pan-cancer dataset with 15 genes which form 105 triplets; we also used different triplet numbers (105, 1,225, and 4,950) on a small subset of the pan-cancer dataset with 250 samples (S4 Fig and S13 Table). Although the ceRNA algorithm was not fast with a large sample size and a large number of triplets compared with SPONGE, GDCRNATools and CERNIA, it was slightly faster than JAMI.’

(line 238-266) ‘Recently, more and more tools have been developed to identify potential ceRNA events. It is important to systematically evaluate the ceRNAR package in comparison with the five other published ceRNA prediction tools—SPONGE [20], CERNIA [21], GDCRNATools [22], JAMI [23], and CUPID [24]—that are expression-based (rather than sequence-based, i.e., spongeScan [25]). We have compared them in terms of several features, such as miRNA-target data sources, study design, ceRNA classes, ceRNA prediction algorithm, and language for implementation (S17 Table). Noting that JAMI is the multi-threading version of CUPID, we decided to keep only JAMI for further analyses. Thus, we used four algorithms, including SPONGE, CERNIA, GDCRNATools, and JAMI, for the comparisons. Notably, the four algorithms and our ceRNAR all used a similar strategy, which is utilizing miRNA-target data sources to identify potential miRNA-gene/lncRNA pairs from other databases. All five of these algorithms are implemented in R and consider the correlations between miRNA-mediated genes/lncRNA. It is worth mentioning that CERNIA and JAMI consider both MRE- and expression-based data, whereas SPONGE, GDCRNATools and ceRNAR only analyze genome-wide expression data. Although these five algorithms are all correlation-based, some differences still exist. For examples, JAMI is based on conditional mutual information, which is particularly useful to capture non-linear associations by estimating the effect of a miRNA on its target pairs through a permutation test [26], while GDCRNATools is based on sensitivity correlation computation through effectively estimating covariate matrices and also considering the impact of a miRNA on its target pairs. SPONGE also uses sensitivity correlation to quantify the impact of a miRNA on its target pairs (i.e., linear partial correlation), but further applies a null model-based p-value computation to estimate potential ceRNA pairs. Since several studies [18, 27, 28] indicate that ceRNA triplets may be observed in a specific range of miRNA expression, such an approach can help to focus on the true positive region with high signal-to-noise ratio instead of missing the ceRNA triplets due to signal dilution by global noise. Our ceRNAR algorithm showed the highest sensitivity in identifying potential ceRNA triplets (Fig 3).’

**Fig 3. Performance comparisons between ceRNIA, JAMI, SPONGE, GDCRNATools and ceRNAR packages using synthetic data that mimic the real world case.** (A) Sensitivity and PPV of various tools at different correlation levels for 100 samples of 105 pairs of target genes under four scenarios. (B) Specificity and NPV of various tools at different correlation levels for 100 sample of 105 pairs of target genes under null scenarios.

**S4 Fig. Run time comparisons between published tools and our packages.** (A) Run time for different sample numbers on a fixed set of genes using real data. (B) Run time for different numbers of genes (i.e., different numbers of triplets) on a fixed number of samples using real data.

**S17 Table. Comparisons with the state-of-the-art tools.**

| **Features** | **ceRNAR** | **SPONGE** | **GDCRNATools** | **CERNIA** | **JAMI** | **Cupid** |
| --- | --- | --- | --- | --- | --- | --- |
| **miRNA-targets data sources** | miRTarBase, miRecords, DIANA-micro T-CDS, EIMMO, miRDB, miRanda, PITA, RNA22, TargetScan | TargetScan v6.0, miRcode | StarBase v2.0, miRcode,  spongScan | miRTarBase, starBase,  miRecords, miRanda, DT-Hybrid | miRecords, TarBase,  TRANSFAC | miRecords, TarBase,  TRANSFAC |
| **TCGA expression profile** | Yes | Yes | Yes | Yes | Yes | Yes |
| **Study design** | Case-only | Case-only | Case-only | Case-only | Case-only | Case-only |
| **ceRNA classes** | miRNA-mRNA | miRNA-mRNA/lncRNA | miRNA-lncRNA | miRNA-mRNA/ | miRNA-mRNA | miRNA-mRNA |
| **ceRNA prediction algorithm** | Pearson correlation +  Data augment + Running sum statistics | Sensitivity correlation for  more than one miRNA | Hypergeometric test +  Pearson correlation +  Regulation similarity | MRE-based +  Gene-expression correlation +  SVM classification | Non-parameter + Multi-threading version of Cupid | MRE-based +  Conditional mutual information |
| **P-value on ceRNA Interaction score** | Yes | Yes | No | No | Yes | Yes |
| **Implementation** | R language | R language | R language | R language | Java, R language | Matlab |
| **Genome-wide screening** | Yes | Yes | Yes | Yes | Yes | Yes |
| **Reference** | N/A | M. List et al. 2019 | R. Li et al. 2018 | D. S. Sardina et al. 2017 | A. Hornakova et al. 2018 | H. Chiu et al. 2014 |

- The output of ceRNAR should be better described, especially the graphic outputs briefly seen in Fig 4
 We appreciate this comment and have presented this figure in four individual supplementary figures (S12-15 Fig) and Fig 4 in the text.

**Fig 4. An overview of our ceRNAR package.** This package includes three main parts. First, “Input data” has three functions: *ceRNAputativePairs()* for extracting curated miRNA-target lists, *ceRNATCGA()* for retrieving TCGA data, and *ceRNAcustomize()* for importing customized data. Second, “Identification of miRNA-ceRNA triplets” involves *ceRNApairFiltering()* and *SegmentClusteringPlusPeakMerging()* and is wrapped into a *ceRNAmethod()* function. Lastly, “Downstream analyses” contains six functions for different tasks, that is, *ceRNAFunction()*, *ceRNAModule()*, *ceRNASurvival()*, *ceRNALocation(), ceRNAValidate() and ceRNAIntegrate()*.

**S12 Fig. Main tabular output in CSV format.** Five columns are involved: miRNA name, candidate ceRNA pairs, the start and end of each miRNA expression interval, and the number of segments.

**S13 Fig. The graphical bubble outputs of the *ceRNAFunctional* function are based on over-representation analysis (ORA).** (A) Based on the KEGG database. (B) Based on the ‘cellular component’ (CC) categories in the gene ontology (GO) database. (C) Based on the ‘molecular function’ (MF) categories in the GO database. (D) Based on the ‘biological process’ (BP) categories in the GO database.

**S14 Fig. The graphical bar outputs of the *ceRNAFunctional* function based on over-representation analysis (ORA).** (A) Based on the KEGG database. (B) Based on the gene ontology (GO) database and stratified by three GO levels: cellular component (CC), molecular function (MF), and biological process (BP).

**S15 Fig. The graphical outputs of the *ceRNAModule*, *ceRNASurvial*, and *ceRNALocation* functions.** (A) Network analysis among candidate ceRNAs for a specific miRNA. (B) Survival analysis for a candidate ceRNA pair targeted by a specific miRNA. (C) A mix of a box plot and bar plot represents the proportion of all candidate ceRNA pairs targeted by a specific miRNA at their corresponding miRNA expression range.

Minor remarks:

Table 1 is reffered to TCGA datasets, not to the simulation dataset. Please fix it
 We apologize for the typo and have revised the description.

(line 675) ‘S14 Table. Summary of the application to the TCGA lung cancer datasets.’

Reviewer #2: The authors propose a computational pipeline to identify ceRNA-miRNA triplets. Specifically, the idea tested here is based on (a) sorting the datapoints based on the miRNA expression level and (b) computing the correlation between expression of genes using the same sorting. This implies a linear relationship between ceRNAs but does not make any assumption on the dependence of the ceRNAs expression level as a function of the miRNA expression level. At the second stage, a segment clustering method is used, which takes into account the ranked miRNA expression levels. Finally, a peak merging is performed to remove false positives. The method is tested on a synthetic dataset and then applied to a dataset taken from the cancer genome atlas.

The discussed approach is interesting since it promises to be much faster than other approaches proposed in the literature. In addition, the authors provide an implementation. However, the manuscript is poorly readable and it is very difficult to appreciate the performance of this method compared with other approaches. I thus think publication is premature at this stage.

We appreciate the comment and have sent the manuscript to a native English speaker for proofreading. We have uploaded a certificate from her.

What is crucially missing are (a) comparisons of the reported method with other methods, perhaps done on the synthetic dataset, and (b) a fair assessment of the accuracy of the method (PPV and sensitivity) on the real dataset. In the current form, it is difficult to see the advantage of this method with respect to competitors.
 We appreciate the comments and have added this in the Results and Discussion sections. We have systematically evaluated five published ceRNA prediction tools, including SPONGE, CERNIA, GDCRNATools, and CUPID / JAMI, in terms of many different features, including miRNA-target data sources, suitable study design, prediction algorithm, and implementation language (S17 Table). Also, we used a synthetic expression dataset generated from a multivariate normal distribution with a randomly selected mean value, ranging from -2 to 2 (±2, ±1, and 0), and a covariance matrix whose entries were also randomly selected from 0.3 to 0.9 (correlation level: all). At the same time, the expression distribution of the uncorrelated genes was specified by setting its mean, which is also randomly selected, to ±2, ±1, and 0; and its variance, also randomly selected, from 0 to 0.2. These settings were based on the sample distribution observed from the TCGA pan-cancer atlas (S16A-C Fig). To construct the covariance matrix, we also specified three correlation levels (high, mid, and low) by randomly selecting from 0.8-0.9, 0.5-0.7, and 0.3-0.4, respectively. Our results (Fig 3) illustrated that the ceRNAR workflow generally outperformed the other tools in terms of sensitivity and PPV in all scenarios when the window size was set to 10 and the correlation cutoff was set to 0.7. GDCRNATools generally had a higher sensitivity (except at higher correlation levels) and lower specificity than ceRNAR, suggesting that it is good for catching ceRNAs but comes with a relatively high rate of false positives. This was mainly observed when the correlation values among correlated genes were relatively high. But all of the tools except JAMI could identify valid ceRNA triplets without reporting high false-positive results. We also used different sample numbers and triplet numbers on a small subset of the pan-cancer dataset to evaluate whether our running time was comparable to other tools. The ceRNAR algorithm is not the most computationally efficient tool, but it is still slightly faster than JAMI (S4 Fig).

(line 155-171) ‘We have compared our tool with other state-of-the-art tools, including SPONGE, JAMI, GDCRNATools, and CERNIA, in terms of their performances using synthetic data and their running time using real data. Fig 3A and S12 Table illustrate that ceRNAR workflow generally outperformed the other tools in terms of sensitivity and PPV in all scenarios when the window size is set to 10 and the correlation cutoff is set to 0.7. It can be mainly observed when the correlation values among correlated genes are relatively high. However, all of the tools could identify valid ceRNA triplets without reporting high false-positive results except JAMI (Fig 3B and S12 Table). GDCRNATools generally had a high sensitivity compared to the other tools, and a lower specificity than ceRNAR, suggesting that it is good for catching ceRNAs but comes with a relatively high rate of false positives. Regarding running time, we used different sample numbers (250, 500, and 100) on a small subset of the pan-cancer dataset with 15 genes which form 105 triplets; we also used different triplet numbers (105, 1,225, and 4,950) on a small subset of the pan-cancer dataset with 250 samples (S4 Fig and S13 Table). Although the ceRNA algorithm was not fast with a large sample size and a large number of triplets compared with SPONGE, GDCRNATools and CERNIA, it was slightly faster than JAMI.’

**Fig 3. Performance comparisons between ceRNIA, JAMI, SPONGE, GDCRNATools and ceRNAR packages using synthetic data that mimic the real world case.** (A) Sensitivity and PPV of various tools at different correlation levels for 100 samples of 105 pairs of target genes under four scenarios. (B) Specificity and NPV of various tools at different correlation levels for 100 sample of 105 pairs of target genes under null scenarios.

**S4 Fig. Run time comparisons between published tools and our packages.** (A) Run time for different sample numbers on a fixed set of genes using real data. (B) Run time for different numbers of genes (i.e., different numbers of triplets) on a fixed number of samples using real data.

**S17 Table. Comparisons with the state-of-the-art tools.**

| **Features** | **ceRNAR** | **SPONGE** | **GDCRNATools** | **CERNIA** | **JAMI** | **Cupid** |
| --- | --- | --- | --- | --- | --- | --- |
| **miRNA-targets data sources** | miRTarBase, miRecords, DIANA-micro T-CDS, EIMMO, miRDB, miRanda, PITA, RNA22, TargetScan | TargetScan v6.0, miRcode | StarBase v2.0, miRcode,  spongScan | miRTarBase, starBase,  miRecords, miRanda, DT-Hybrid | miRecords, TarBase,  TRANSFAC | miRecords, TarBase,  TRANSFAC |
| **TCGA expression profile** | Yes | Yes | Yes | Yes | Yes | Yes |
| **Study design** | Case-only | Case-only | Case-only | Case-only | Case-only | Case-only |
| **ceRNA classes** | miRNA-mRNA | miRNA-mRNA/lncRNA | miRNA-lncRNA | miRNA-mRNA/ | miRNA-mRNA | miRNA-mRNA |
| **ceRNA prediction algorithm** | Pearson correlation +  Data augment + Running sum statistics | Sensitivity correlation for  more than one miRNA | Hypergeometric test +  Pearson correlation +  Regulation similarity | MRE-based +  Gene-expression correlation +  SVM classification | Non-parameter + Multi-threading version of Cupid | MRE-based +  Conditional mutual information |
| **P-value on ceRNA Interaction score** | Yes | Yes | No | No | Yes | Yes |
| **Implementation** | R language | R language | R language | R language | Java, R language | Matlab |
| **Genome-wide screening** | Yes | Yes | Yes | Yes | Yes | Yes |
| **Reference** | N/A | M. List et al. 2019 | R. Li et al. 2018 | D. S. Sardina et al. 2017 | A. Hornakova et al. 2018 | H. Chiu et al. 2014 |

Figure 2 reports results for the synthetic dataset. It is not clear from this figure how the critical parameters (windows size and thresholds) can be optimized in absence of a reference result. Indeed, as fairly written in the discussion, the choice of these parameters in real applications is still to be fine tuned.
 We appreciate the comments and have revised this section. We have re-analyzed the real data from the TCGA dataset. The real dataset was retrieved from the TCGA pan-cancer atlas (https://gdc.cancer.gov/about-data/publications/pancanatlas), and it was first log-transformed and then transformed into z score by z transformation, which allows the expression values to be comparable among disparate distributions. After such data processing, the sample distribution of a gene generally tends to be a bell-shaped distribution with a specific mean (S16A&B Fig). Therefore, in the new simulation study, we allowed data sampling from a multivariate normal distribution with different means in order to reflect the real dataset, which was randomly selected from -2 to 2, and different covariance matrices contained various correlation values (randomly selected from 0.3 to 0.9 for correlated genes and from 0 to 0.2 for uncorrelated genes) (S16A-C Fig). We also provided the optimal parameter setting for this real data. For 100 samples, the best window size was 10, and the best cutoff correlation value for selecting the most significant event was 0.7 (S3 Fig).

(line 511-520) ‘Another simulation scenario is mimicking a real-data profile generated from a multivariate normal distribution with a randomly selected mean value (±2, ±1 and 0), and a covariance matrix whose entries are also randomly selected from 0.3 to 0.9 (correlation level: all), while the expression distribution of the uncorrelated genes was specified by setting its mean (also randomly selected) at ±2, ±1, and 0, and its variance (randomly selected) at 0 to 0.2 (S16 Fig). We also specified three correlation levels (high = 0.8-0.9, mid = 0.5-0.7, and low = 0.3-0.4) and randomly selected values within them to construct the covariance matrix. The normality test for sample distribution of each gene was conducted by the Anderson-Darling method [53].’

(line 130-152) ‘In addition, to ensure the algorithm only sensitively identified ceRNA events under higher correlation values between them, we also generated simulated data from a multivariate normal distribution with the same mean value but a covariance matrix whose entries are 0.6 (S2A Fig and S6 Table) or 0.3 (S2B and S7 Table). Although the ceRNAR algorithm was still able to detect ceRNA pairs with moderate sensitivity values (0.4 to 0.5) when the correlation values between target genes within a pair were 0.6, it did not work well (sensitivity values were all below 0.25) when the correlation values between target genes within a pair were 0.3. Together, these results support our correlation-based hypothesis that ceRNA events tend to occur when they are highly correlated. However, the above-mentioned results were based on simple and naïve simulations. We also mimicked the distribution of expression from pan-cancer data (9,835 samples), allowing the synthetic data to be generated from a multivariate normal distribution with a randomly selected mean value and a covariance matrix whose entries are also randomly selected. The simulation studies were also performed under different correlation levels. As shown in S3 Fig and S8-S11 Tables, the performance of the ceRNAR algorithm was determined by the level of the correlation values. Nevertheless, it still performed well (sensitivity values > 0.75) when the correlation between genes was high and when the window size was 10 throughout all scenarios (S3B Fig.), suggesting the ceRNAR algorithm is efficient to identify most potential ceRNA pairs when their correlation pattern is relatively high (0.8-0.9) to compete with a specific miRNA, and such a pattern exists in at least 20% of the sample. The optimal parameter settings for real data were also observed. For 100 samples, the best window size was 10, and the best cutoff correlation value for selecting the most significant event was 0.7.’

**S16 Fig. Summary statistics of the gene expression profile of 9,835 samples in TCGA pan-cancer atlas.** (A) The density plots represent the sample distribution of observed z-scores of 10 genes. (B) The density plot and boxplot represent the sample distribution of observed z-scores of 16,323 genes. (C) The pie chart shows the proportion of the Pearson correlation values of 16,323 genes. All the expression values are log-transformed and z-transformed across cancers. The median expression values of samples across cancers are used for summary statistics.

**S3 Fig. The performance of our proposed method in four scenarios (I to IV) using simulated data that mimics real-world data distribution with the complete version.** (A) From a covariance matrix with correlation values ranging from 0.3 to 0.9. (B) From a covariance matrix with correlation values ranging from 0.8 to 0.9. (C) From a covariance matrix with correlation values ranging from 0.5 to 0.7. (D) From a covariance matrix with correlation values ranging from 0.3 to 0.4.

The comparison with other identified triplets is merely anecdotal, with a quick mention of two previously reported triplets, but there is no systematic analysis performed. In addition, the authors should compare their method with alternative methods on their synthetic datasets, providing a quantitative assessment on the performance difference (both in terms of computational cost and quality of the results).
 We appreciate the comments and have revised this section. We have added an R function, *ceRNValidate()*, for external validation of the findings identified by our algorithm using experimentally validated miRNA and its target interactions collected in miRsponge. We also used graphic outputs to summarize those experimentally validated results supporting the potential ceRNA events identified by the ceRNAR algorithm (S7 Fig). As shown in S7 Fig, around 1% of experimentally validated ceRNA triplets were identified in LUAD and LUSC. The low proportion may be attributed to the fact that only 158 ceRNA triplets were analyzed and that those ceRNA triplets may not be expressed in lung tissues. Furthermore, approximately 13-14% of ceRNA triplets with at least one experimentally validated miRNA-target interaction were identified by using the ceRNAR algorithm. A Chi-square test was performed to examine whether the findings from the ceRNAR package were significantly enriched in the database, and the results showed that the P-values obtained from the two datasets, LUAD and LUSC, were both less than 2.2e-16, suggesting our ceRNAR package can successfully identify previously reported ceRNA triplets. Regarding a quantitative assessment of the performance, we have compared our method with published methods using our synthetic dataset and provided a quantitative assessment of the performance (sensitivity, specificity, PPV, and NPV) (Fig 3). We also did a running time comparison using real data in terms of different sample numbers and triplet numbers (S4 Fig). Although ceRNAR may not be the most computationally efficient tool, the performance is better than other tools.

(line 496-499) ‘We also implemented an integrated function (*ceRNAIntegrate*) to combine our results with other state-of-the-art tools, such as SPONGE [20] and JAMI [23], and a validation function (*ceRNAValidate*) based on the miRSponge database.’

(line 200-212) ‘In addition, we compared our findings against experimentally validated miRNA-gene pairs in the miRSponge database to endorse the potential ceRNAs identified by ceRNAR. As shown in S7 Figure, around 1% (ceRNA pairs that can be validated among total ceRNA founded based on ceRNAR algorithm) of experimentally validated ceRNA triplets were identified in LUAD and LUSC. The low proportion may be attributed to the fact that only 158 ceRNA triplets were analyzed and that those ceRNA triplets may not be expressed in lung tissues. Furthermore, approximately 13-14% of ceRNA triplets with at least one experimentally validated miRNA-target interaction were identified by using the ceRNAR algorithm. A Chi-square test was performed to examine whether the findings from the ceRNAR package were significantly enriched in the TCGA data, and the results showed that the P-values obtained from the LUAD and LUSC datasets were both less than 2.2e-16, suggesting our ceRNAR package can successfully identify previously reported ceRNA triplets.’

(line 155-171) ‘We have compared our tool with other state-of-the-art tools, including SPONGE, JAMI, GDCRNATools, and CERNIA, in terms of their performances using synthetic data and their running time using real data. Fig 3A and S12 Table illustrate that ceRNAR workflow generally outperformed the other tools in terms of sensitivity and PPV in all scenarios when the window size is set to 10 and the correlation cutoff is set to 0.7. It can be mainly observed when the correlation values among correlated genes are relatively high. However, all of the tools could identify valid ceRNA triplets without reporting high false-positive results except JAMI (Fig 3B and S12 Table). GDCRNATools generally had a high sensitivity compared to the other tools, and a lower specificity than ceRNAR, suggesting that it is good for catching ceRNAs but comes with a relatively high rate of false positives. Regarding running time, we used different sample numbers (250, 500, and 100) on a small subset of the pan-cancer dataset with 15 genes which form 105 triplets; we also used different triplet numbers (105, 1,225, and 4,950) on a small subset of the pan-cancer dataset with 250 samples (S4 Fig and S13 Table). Although the ceRNA algorithm was not fast with a large sample size and a large number of triplets compared with SPONGE, GDCRNATools and CERNIA, it was slightly faster than JAMI.’

(line 238-266) ‘Recently, more and more tools have been developed to identify potential ceRNA events. It is important to systematically evaluate the ceRNAR package in comparison with the five other published ceRNA prediction tools—SPONGE [20], CERNIA [21], GDCRNATools [22], JAMI [23], and CUPID [24]—that are expression-based (rather than sequence-based, i.e., spongeScan [25]). We have compared them in terms of several features, such as miRNA-target data sources, study design, ceRNA classes, ceRNA prediction algorithm, and language for implementation (S17 Table). Noting that JAMI is the multi-threading version of CUPID, we decided to keep only JAMI for further analyses. Thus, we used four algorithms, including SPONGE, CERNIA, GDCRNATools, and JAMI, for the comparisons. Notably, the four algorithms and our ceRNAR all used a similar strategy, which is utilizing miRNA-target data sources to identify potential miRNA-gene/lncRNA pairs from other databases. All five of these algorithms are implemented in R and consider the correlations between miRNA-mediated genes/lncRNA. It is worth mentioning that CERNIA and JAMI consider both MRE- and expression-based data, whereas SPONGE, GDCRNATools and ceRNAR only analyze genome-wide expression data. Although these five algorithms are all correlation-based, some differences still exist. For examples, JAMI is based on conditional mutual information, which is particularly useful to capture non-linear associations by estimating the effect of a miRNA on its target pairs through a permutation test [26], while GDCRNATools is based on sensitivity correlation computation through effectively estimating covariate matrices and also considering the impact of a miRNA on its target pairs. SPONGE also uses sensitivity correlation to quantify the impact of a miRNA on its target pairs (i.e., linear partial correlation), but further applies a null model-based p-value computation to estimate potential ceRNA pairs. Since several studies [18, 27, 28] indicate that ceRNA triplets may be observed in a specific range of miRNA expression, such an approach can help to focus on the true positive region with high signal-to-noise ratio instead of missing the ceRNA triplets due to signal dilution by global noise. Our ceRNAR algorithm showed the highest sensitivity in identifying potential ceRNA triplets (Fig 3).’

**Fig 4. An overview of our ceRNAR package.** This package includes three main parts. First, “Input data” has three functions: *ceRNAputativePairs()* for extracting curated miRNA-target lists, *ceRNATCGA()* for retrieving TCGA data, and *ceRNAcustomize()* for importing customized data. Second, “Identification of miRNA-ceRNA triplets” involves *ceRNApairFiltering()* and *SegmentClusteringPlusPeakMerging()* and is wrapped into a *ceRNAmethod()* function. Lastly, “Downstream analyses” contains six functions for different tasks, that is, *ceRNAFunction()*, *ceRNAModule()*, *ceRNASurvival()*, *ceRNALocation(), ceRNAValidate() and ceRNAIntegrate()*.

**S7 Fig. Experimental validation of real case study based on miRSponge database.** (A) TCGA-LUAD (The Cancer Genome Atlas Lung Adenocarcinoma). (B) TCGA-LUSC (The Cancer Genome Atlas Lung Squamous Cell Carcinoma).

**Fig 3. Performance comparisons between ceRNIA, JAMI, SPONGE, GDCRNATools and ceRNAR packages using synthetic data that mimic the real world case.** (A) Sensitivity and PPV of various tools at different correlation levels for 100 samples of 105 pairs of target genes under four scenarios. (B) Specificity and NPV of various tools at different correlation levels for 100 sample of 105 pairs of target genes under null scenarios.

**S4 Fig. Run time comparisons between published tools and our packages.** (A) Run time for different sample numbers on a fixed set of genes using real data. (B) Run time for different numbers of genes (i.e., different numbers of triplets) on a fixed number of samples using real data.

Finally, the text should be better proof read. There are a number of incorrect sentences that makes it difficult to read. E.g., "there is still needed for a tool" (abstract) "simulations of different parameters" (line 134; perhaps "simulations with different parameters"?). Some acronyms are defined after they are use and also makes the paper difficult to read.

We appreciate the comment and have sent the manuscript to a native English speaker for proofreading. We have also uploaded a certificate from her.

Reviewer #3: With ceRNAR, Hsiao et al. present a new R package for inferring competing endogenous RNA triplets from paired gene and miRNA expression data. In contrast to prior approaches that leverage conditional mutual information or partial correlation to assess the mutual influence that ceRNAs exert on each other via miRNA binding competition, ceRNAR considers a direct correlation approach for which miRNAs with similar expression are grouped in windows. The likely intention behind this is to fix the miRNA expression to then investigate the competition in absence of miRNA changes. I have the following comments:

#Major:

- The basic intuition of the method is not explained in sufficient detail. Only the sentence "we consider gene expression and known interactions among miRNA-gene pairs at a specific miRNA expression level" point to this but does not explain the rationale behind this approach. It is left to the reader to reverse engineer the underlying hypothesis from the method section.

We apologize that we did not clearly explain the rationale of our approach and have revised the Materials and Methods section. We have two assumptions in this study: (1) the expression levels of two target genes tend to be highly correlated when a possible ceRNA event occurs; (2) such events between target genes of a certain miRNA occur at a specific expression interval of that miRNA. However, for each ceRNA triplet from the real data, it is difficult to know which levels of miRNA expression (low, middle, or high expression intervals) will lead to a high correlation between a pair of target genes. To address this issue, we adopted the sliding window approach to identify the correlation patterns of the two target genes within a specific range of expression values for the miRNA. That is the reason why we ranked the samples based on their miRNA expression value from each triplet to identify the correlation patterns and provided the number of samples that meet the criteria.

- While the idea is certainly interesting, I'm not convinced that fixing a miRNA expression level works the way the authors expect. If the idea is to remove the influence of miRNAs one has to consider that miRNA expression changes are dynamic. Several scenarios are plausible for observing a given miRNA expression level. For instance, the miRNA might have been upregulated together with a ceRNA which then compensates for the extra miRNA copies via miRNA binding / sponging. The authors should describe in detail in which scenarios their method is likely to be successful and where it is not.

We appreciate the suggestion and totally agree with the comment that the basic assumption of the approach used in this study may not fit all scenarios in the real data. Definitely, the expression level of one miRNA can be regulated by many factors, such as compensation and/or other interactors. However, with our current understanding of all miRNAs, it is not feasible to consider all potential regulators of one specific miRNA at the same time. Therefore, for one single miRNA, we adapted the approach of utilizing its expression level as the final output instead of considering all possible confounding factors, which can be regarded as the hidden layers of the miRNA expression value. We have revised the Discussion section to address this issue in the manuscript. Lastly, to make our basic assumptions clearer, we added the following sentences into the manuscript. As described in the response to comment 1 from Reviewer 3, the idea behind our approach is to consider the influence of one miRNA through the ranking of the samples based on their miRNA expression level. We have two assumptions in this study: (1) two target genes tend to be highly correlated when a possible ceRNA event occurs; (2) such events between target genes of a certain miRNA occur at a specific expression interval of that miRNA. Specifically, we can visualize the correlation values between target genes and ranked miRNA expression using a dot plot (S10 Fig). Notably, for one miRNA, the high correlation values between two target genes can only be observed in a specific range of the miRNA expression.

**S10 Fig. A graphic overview illustrates the random walk and permutation test in the *ceRNApairFiltering* method.** First, the k correlation values per triplet are ordered according to the average miRNA expression, ${miRNA}_{k}={\sum_{n=0}^{w-1} m_{k+n}^{m,i,j}}/w$. The score (S) reflects the degree to which the correlation of a gene pair is overrepresented across all values (i.e., high/low/moderate miRNA expression) of the entire set of ranked miRNA expression levels. It is calculated by walking down the ranked value, increasing the score when encountering a specific miRNA expression value with the correlation of a paired gene over 0.3. If the correlation is less than 0.3 (threshold), the score will be negative. The magnitude of the score depends on the number of samples supported by such correlation values within a window. Subsequently, the permuted miRNA expression data are generated 1,000 times to create a null distribution of the S, and then the empirical P-value is calculated accordingly.

- The manuscript describes two hypotheses (i) miRNA tends to be sequestered by the miRNA with the higher expression level and (ii) miRNA has a greater affinity for the mRNA with more MRE. It should be highlighted that likely both effects are relevant and mixed.

We appreciate this suggestion and have revised the sentence accordingly. The original idea of the first hypothesis is based on the condition that the number of MREs is the same between two target genes of a specific miRNA.

(line24-25) ‘…under the assumption of equal MREs on the two RNAs.’

- The manuscript does not describe the state of the art sufficiently. Several tools such as CUPID / JAMI or SPONGE are missing here.

We appreciate this comment and have added the comparison with the state-of-the-art tools in the Results and Discussion sections. We have systematically evaluated five published ceRNA prediction tools, including SPONGE, CERNIA, GDCRNATools, and CUPID / JAMI, in terms of many different features, including miRNA-target data sources, suitable study design, prediction algorithm, and implementation language (S17 Fig). Note that JAMI is the multi-threading version of CUPID and thus we decided to evaluate JAMI rather than CUPID. These tools and our tool all have used miRNA-target data sources to focus on the potential miRNA-gene/lncRNA that have been validated or predicted in other databases. All of them are implemented in R and apply the concept of correlation between miRNA-mediated genes/lncRNA. However, CERNIA and JAMI use both MRE- and expression-based data, but SPONGE and our tool only rely on genome-wide expression data. We used different sample numbers and triplet numbers on a small subset of the pan-cancer dataset to evaluate whether our running time is comparable to other tools. The ceRNA algorithm is not the most computationally efficient tool, but it is still slightly faster than JAMI (S4 Fig).

(line 165-171) ‘Regarding running time, we used different sample numbers (250, 500, and 100) on a small subset of the pan-cancer dataset with 15 genes which form 105 triplets; we also used different triplet numbers (105, 1,225, and 4,950) on a small subset of the pan-cancer dataset with 250 samples (S4 Fig and S13 Table). Although the ceRNA algorithm was not fast with a large sample size and a large number of triplets compared with SPONGE, GDCRNATools and CERNIA, it was slightly faster than JAMI.’

(line 238-266) ‘Recently, more and more tools have been developed to identify potential ceRNA events. It is important to systematically evaluate the ceRNAR package in comparison with the five other published ceRNA prediction tools—SPONGE [20], CERNIA [21], GDCRNATools [22], JAMI [23], and CUPID [24]—that are expression-based (rather than sequence-based, i.e., spongeScan [25]). We have compared them in terms of several features, such as miRNA-target data sources, study design, ceRNA classes, ceRNA prediction algorithm, and language for implementation (S17 Table). Noting that JAMI is the multi-threading version of CUPID, we decided to keep only JAMI for further analyses. Thus, we used four algorithms, including SPONGE, CERNIA, GDCRNATools, and JAMI, for the comparisons. Notably, the four algorithms and our ceRNAR all used a similar strategy, which is utilizing miRNA-target data sources to identify potential miRNA-gene/lncRNA pairs from other databases. All five of these algorithms are implemented in R and consider the correlations between miRNA-mediated genes/lncRNA. It is worth mentioning that CERNIA and JAMI consider both MRE- and expression-based data, whereas SPONGE, GDCRNATools and ceRNAR only analyze genome-wide expression data. Although these five algorithms are all correlation-based, some differences still exist. For examples, JAMI is based on conditional mutual information, which is particularly useful to capture non-linear associations by estimating the effect of a miRNA on its target pairs through a permutation test [26], while GDCRNATools is based on sensitivity correlation computation through effectively estimating covariate matrices and also considering the impact of a miRNA on its target pairs. SPONGE also uses sensitivity correlation to quantify the impact of a miRNA on its target pairs (i.e., linear partial correlation), but further applies a null model-based p-value computation to estimate potential ceRNA pairs. Since several studies [18, 27, 28] indicate that ceRNA triplets may be observed in a specific range of miRNA expression, such an approach can help to focus on the true positive region with high signal-to-noise ratio instead of missing the ceRNA triplets due to signal dilution by global noise. Our ceRNAR algorithm showed the highest sensitivity in identifying potential ceRNA triplets (Fig 3).’

**S4 Fig. Run time comparisons between published tools and our packages.** (A) Run time for different sample numbers on a fixed set of genes using real data. (B) Run time for different numbers of genes (i.e., different numbers of triplets) on a fixed number of samples using real data.

**S17 Table. Comparisons with the state-of-the-art tools.**

| **Features** | **ceRNAR** | **SPONGE** | **GDCRNATools** | **CERNIA** | **JAMI** | **Cupid** |
| --- | --- | --- | --- | --- | --- | --- |
| **miRNA-targets data sources** | miRTarBase, miRecords, DIANA-micro T-CDS, EIMMO, miRDB, miRanda, PITA, RNA22, TargetScan | TargetScan v6.0, miRcode | StarBase v2.0, miRcode,  spongScan | miRTarBase, starBase,  miRecords, miRanda, DT-Hybrid | miRecords, TarBase,  TRANSFAC | miRecords, TarBase,  TRANSFAC |
| **TCGA expression profile** | Yes | Yes | Yes | Yes | Yes | Yes |
| **Study design** | Case-only | Case-only | Case-only | Case-only | Case-only | Case-only |
| **ceRNA classes** | miRNA-mRNA | miRNA-mRNA/lncRNA | miRNA-lncRNA | miRNA-mRNA/ | miRNA-mRNA | miRNA-mRNA |
| **ceRNA prediction algorithm** | Pearson correlation +  Data augment + Running sum statistics | Sensitivity correlation for  more than one miRNA | Hypergeometric test +  Pearson correlation +  Regulation similarity | MRE-based +  Gene-expression correlation +  SVM classification | Non-parameter + Multi-threading version of Cupid | MRE-based +  Conditional mutual information |
| **p-value on ceRNA interaction score** | Yes | Yes | No | No | Yes | Yes |
| **Implementation** | R language | R language | R language | R language | Java, R language | Matlab |
| **Genome-wide screening** | Yes | Yes | Yes | Yes | Yes | Yes |
| **Reference** | N/A | M. List et al. 2019 | R. Li et al. 2018 | D. S. Sardina et al. 2017 | A. Hornakova et al. 2018 | H. Chiu et al. 2014 |

- Furthermore, a performance comparison of ceRNAR to these tools which infer ceRNA-miRNA triplets is missing.

We appreciate this comment and have added the comparison with the state-of-the-art tools, including SPONGE, CERNIA, GDCRNATools, and JAMI, in the Results and Discussion sections. We have compared our method with published methods on our synthetic dataset and provided a quantitative assessment of the performance (sensitivity, specificity, PPV, and NPV). Our results (Fig 3) illustrated that the ceRNAR workflow generally outperformed the other tools in terms of sensitivity and PPV in all scenarios when the window size was set to 10 and the correlation cutoff was set to 0.7. This was mainly observed when the correlation values among correlated genes were relatively high. GDCRNATools generally had a higher sensitivity (except at higher correlation levels) and lower specificity than ceRNAR, suggesting that it is good for catching ceRNAs but comes with a relatively high rate of false positives.

(line 155-165) ‘We have compared our tool with other state-of-the-art tools, including SPONGE, JAMI, GDCRNATools, and CERNIA, in terms of their performances using synthetic data and their running time using real data. Fig 3A and S12 Table illustrate that ceRNAR workflow generally outperformed the other tools in terms of sensitivity and PPV in all scenarios when the window size is set to 10 and the correlation cutoff is set to 0.7. It can be mainly observed when the correlation values among correlated genes are relatively high. However, all of the tools could identify valid ceRNA triplets without reporting high false-positive results except JAMI (Fig 3B and S12 Table). GDCRNATools generally had a high sensitivity compared to the other tools, and a lower specificity than ceRNAR, suggesting that it is good for catching ceRNAs but comes with a relatively high rate of false positives.’

**Fig 3. Performance comparisons between ceRNIA, JAMI, SPONGE, GDCRNATools and ceRNAR packages using synthetic data that mimic the real world case.** (A) Sensitivity and PPV of various tools at different correlation levels for 100 samples of 105 pairs of target genes under four scenarios. (B) Specificity and NPV of various tools at different correlation levels for 100 sample of 105 pairs of target genes under null scenarios.

- Most ceRNA pairs share more than one miRNA and should ideally be considered together as e.g. in the SPONGE method. This is not feasible here since we can sort only for one miRNA but the limitation should be discussed.

We appreciate the comment and have discussed this limitation in the Discussion section.

(line 325-329) ‘Therefore, the consideration of all types of miRNA sponges, the amount of MREs, multiple miRNAs that may compete for the same pair of target genes, nontrivial correlations which involve the comparison of pairwise correlation and pairwise partial correlation, and the minimization of computational time are important key areas for further optimization and extension of the ceRNAR algorithm.’

- The method descriptions are not easy enough to understand, e.g. in "The ceRNApair Filtering method" it is not explaind what is meant by "window", i.e window of miRNA expression values in the sorted expression vector.

We apologized for the confusion about the terminology and have revised as follows. The definition of the “window” in a particular dataset is the length of sample size (always less than the total sample size) that is used to calculate the correlation values between two target genes (S9 Fig). To analyze all samples in the dataset, the window can be moved from the first group of samples to the last group of samples gradually (S9 Fig), which is the sliding window approach. Identifying the window with the highest correlation also identifies the specific range of miRNA expression at which the correlation occurs.

(line 397-399) ‘The purpose of this function is to identify ceRNA-miRNA triplets based on the Pearson correlation coefficient through the sliding window approach (S9 Fig) and a running sum statistic for such values by the random walk approach (S10 Fig).’

(line 405-408) ‘Technically, the correlation value between two genes is calculated using the gene expression values of all samples. By using a sliding window approach [45], we can artificially create different varieties of a real dataset to increase its size, which is a sort of data augmentation technique [46].’

**S9 Fig. A graphic overview illustrates sorting and correlation calculation in the *ceRNApairFiltering* method.** First, samples based on the expression vector of each miRNA per triplet (genei, miRNAm, genej) are sorted. Second, the window size is defined, and the correlation between gene pairs among samples for the first window is calculated. Next, the correlation between gene pairs among samples for the second window is calculated, and so on. Finally, a new correlation vector per triplet is created.

- The whole intuition behind Pobey and Pviolate is lost to me. This makes it hard to understand what the method aims to show here. Please revise this section.

We apologize for the confusion and have revised this section thoroughly. We followed the concept of the Gene Set Enrichment Analysis (GSEA) algorithm [1], which captures the accumulated changes in the expression of all genes within a pathway through a random-walk method. The main idea of the GSEA algorithm is to understand whether the differentially expressed genes are significantly enriched in the samples belonging to the same phenotype (case or control). First, the differentially expressed genes were ranked by using the differences in expression level between the two phenotypes. Next, the GSEA algorithm gave a positive score to the genes located in the pathway, whereas a negative score was assigned to the genes outside of the pathway. One possible scenario to obtain a high score is that the differentially expressed genes were clustered and enriched in one phenotype instead of being randomly distributed. This scenario is the same as what we want to identify for the ceRNA triplet. That is, the highest correlation values were observed in a specific range of the miRNA expression and thus we used the two statistics, P_obey_ and P_violate_ to identify the range (S11 Fig). The definition of the P_obey_ is the proportion of samples whose gene correlations are over the threshold (default value is 0.3, i.e. $r_{k}^{m,i,j}>0.3$), and the definition of P_violate_ is the proportion of the rest of samples at a given ranking window position k across all samples. Subsequently, the score S was calculated based on the values of P_obey_ and P_violate_ using the formula shown in S11 Fig. For one miRNA, a low score S will be observed if the two target genes have no correlation within a specific range of miRNA expression. On the other hand, the score S will be high if a large proportion of the samples showed high correlation among the two target genes (P_obey_ is high). Consequently, we can use the score S to identify a ceRNA triplet when S is high, and a statistical approach was performed to ensure that S cannot be identified randomly.

[1] Subramanian A, Tamayo P, Mootha VK, Mukherjee S, Ebert BL, Gillette MA, et al. Gene set enrichment analysis: a knowledge-based approach for interpreting genome-wide expression profiles. Proceedings of the National Academy of Sciences. 2005;102(43):15545-50.

(line 422-433) ‘S is then defined as the maximum distance from zero of the substations of P_obey_(k) from P_violate_(k). If the samples with similar correlation values are not enriched at a particular miRNA expression interval, it means those gene pairs are not biologically relevant to compete with miRNA in a miRNA-gene triplet; that is, there is no ceRNA event observed in this triplet (S11 Fig). Finally, we accessed the significance of an observed S by comparing it with the theoretical S computed by randomly permutating the expression of the candidate miRNA_m_ 1,000 times to provide assessment of significance. When an entire sample of potential triplets is evaluated, these p-values will be adjusted by the false discovery rate, which provides the estimated probability of whether a triplet within an entire set of potential triplets is a false positive finding or not.’

**S11 Fig. The meanings of score S are based on the values of P_obey_ and P_violate_.** (A) Samples with the highest correlation between target genes are enriched at a higher miRNA expression value, supporting that these two target genes have a higher chance to compete with this miRNA. (B) These target gene pairs do not represent a biologically relevant correlation with the competition of this miRNA, suggesting that no ceRNA event occurs in this triplet.

- Similarly, I fail to understand the motivation for the segment clustering and peak merging. What peaks are we talking about?

We apologize for the confusion and have revised this section. The motivation of the segment clustering is to group the samples showing high correlation between the expression levels of the two target genes into one single cluster. In our analysis, we divided the samples into small groups (i.e., a window) to calculate the correlation values of the two target genes. Accordingly, we may identify several different groups showing high correlations that actually can be clustered into one single cluster because of their similar correlation values. So, we performed the segment clustering to evaluate whether those small windows showing high correlation values should be clustered into a larger group. This method has been widely used in the analysis of copy number variations (CNVs) by many algorithms [2-3]. After performing the segment clustering approach, only a few groups of samples showing high correlation remain for further analyses. We have a basic assumption here for the ceRNA triplet: the correlation values should be stable across the sliding window approach and thus the correlation values should not be bouncing up and down within a small sample size. Following this assumption, we performed the peak merging step to ensure two nearby peaks were merged into one under certain criteria.

[2] Tickle T, Tirosh I, Georgescu C, Brown M, Haas B. inferCNV of the Trinity CTAT Project. Klarman Cell Observatory, Broad Institute of MIT and Harvard. 2019.

[3] Wang K, Li M, Hadley D, Liu R, Glessner J, Grant SF, et al. PennCNV: an integrated hidden Markov model designed for high-resolution copy number variation detection in whole-genome SNP genotyping data. Genome research. 2007;17(11):1665-74.

(line 463-466) ‘This method is designed to prevent smashed segments resulting from noise. First, two segments are merged when their distance is smaller than a predefined value, and whether the finalized segments are peaks or troughs is defined compared to the baseline.’

(line 468-474) ‘Two adjacent peaks are further merged if there is no statistically significant difference between them. Because we presumed it is less likely that two genes are highly correlated to compete for a miRNA at more than two miRNA expression intervals, the triplet was abandoned when more than two peaks occurred in such a relationship. Lastly, candidate ceRNAs are finally selected when their correlation pattern contains a peak with a correlation value over a predefined threshold value (0.3, 0.5, or 0.7).’

- Regarding the simulation: why is the mean and variance of uncorrelated genes set to zero? This would mean that no uncorrelated genes are expressed.

We apologize for the confusion and revised the settings accordingly. The original values for the mean and variance of uncorrelated genes were obtained after normalization. This is why we had zero as the setting. However, following the suggestion from the Reviewer 3, we re-analyzed the real data from the TCGA dataset. The real dataset was retrieved from the TCGA pan-cancer atlas (https://gdc.cancer.gov/about-data/publications/pancanatlas), and it was first log-transformed and then transformed into z score by z transformation, which allows the expression values to be comparable among disparate distributions. After such data processing, the sample distribution of a gene tends to generally be a bell-shaped distribution with a specific mean (S16A&B Fig). Therefore, in the new simulation study, we allowed data sampling from a multivariate normal distribution with different means in order to reflect the real dataset, which was randomly selected from -2 to 2, and the different covariance matrices contained various correlation values (randomly selected from 0.3 to 0.9 for correlated genes and from 0 to 0.2 for uncorrelated genes) (S16A-C Fig).

(line 511-520) ‘Another simulation scenario is mimicking a real-data profile generated from a multivariate normal distribution with a randomly selected mean value (±2, ±1 and 0), and a covariance matrix whose entries are also randomly selected from 0.3 to 0.9 (correlation level: all), while the expression distribution of the uncorrelated genes was specified by setting its mean (also randomly selected) at ±2, ±1, and 0, and its variance (randomly selected) at 0 to 0.2 (S16 Fig). We also specified three correlation levels (high = 0.8-0.9, mid = 0.5-0.7, and low = 0.3-0.4) and randomly selected values within them to construct the covariance matrix. The normality test for sample distribution of each gene was conducted by the Anderson-Darling method [53].’

**S16 Fig. Summary statistics of the gene expression profile of 9,835 samples in TCGA pan-cancer atlas.** (A) The density plots represent the sample distribution of observed z-scores of 10 genes. (B) The density plot and boxplot represent the sample distribution of observed z-scores of 16,323 genes. (C) The pie chart shows the proportion of the Pearson correlation values of 16,323 genes. All the expression values are log-transformed and z-transformed across cancers. The median expression values of samples across cancers are used for summary statistics.

- The simulation setting is way too simplistic. Here, the manuscript only assumes a correlation of 0.9 between ceRNA pairs but in reality this can take any value. More realistic would be to set an random correlation of 0.3-0.9 here for each pair. Realistically, gene pairs that are not ceRNAs will also have a meaningful positive correlation which is why typically conditional mutual information or partial correlation needs to be used to determine the miRNA contribution in the triplet. I don't think the simulation results are hence reflecting the performance on real-world data. A more realistic simulation would be needed for a fair method comparison though (see my comment above).

We sincerely appreciate the suggestion and have added several simulation scenarios accordingly. The simulated data was originally sampled from a predefined multivariate normal distribution with a mean of zero and a predefined covariance matrix with a fixed correlation value (0.9). We have redesigned this to make the simulation more flexible. The covariance matrix used in the simulation can be randomly selected from 0.3 to 0.9 (S16C Fig), and the matrix must be symmetric with a positive definite covariance matrix. We followed the suggestion from the reviewer and performed an analysis of the real pan-cancer data from the TCGA (9,835 samples). Notably, the ranges of the gene expression after log and z transformation are around -2 to +2 (S16A&B Fig); as a result, the simulation parameters were changed from the multivariate normal distribution with a predefined mean value to a random number selected from the same interval to ensure our simulation results reflected the performance on real-world data (S2-3 Figs and S8-11 Tables).

(line 130-152) ‘In addition, to ensure the algorithm only sensitively identified ceRNA events under higher correlation values between them, we also generated simulated data from a multivariate normal distribution with the same mean value but a covariance matrix whose entries are 0.6 (S2A Fig and S6 Table) or 0.3 (S2B and S7 Table). Although the ceRNAR algorithm was still able to detect ceRNA pairs with moderate sensitivity values (0.4 to 0.5) when the correlation values between target genes within a pair were 0.6, it did not work well (sensitivity values were all below 0.25) when the correlation values between target genes within a pair were 0.3. Together, these results support our correlation-based hypothesis that ceRNA events tend to occur when they are highly correlated. However, the above-mentioned results were based on simple and naïve simulations. We also mimicked the distribution of expression from pan-cancer data (9,835 samples), allowing the synthetic data to be generated from a multivariate normal distribution with a randomly selected mean value and a covariance matrix whose entries are also randomly selected. The simulation studies were also performed under different correlation levels. As shown in S3 Fig and S8-S11 Tables, the performance of the ceRNAR algorithm was determined by the level of the correlation values. Nevertheless, it still performed well (sensitivity values > 0.75) when the correlation between genes was high and when the window size was 10 throughout all scenarios (S3B Fig.), suggesting the ceRNAR algorithm is efficient to identify most potential ceRNA pairs when their correlation pattern is relatively high (0.8-0.9) to compete with a specific miRNA, and such a pattern exists in at least 20% of the sample. The optimal parameter settings for real data were also observed. For 100 samples, the best window size was 10, and the best cutoff correlation value for selecting the most significant event was 0.7.’

**S16 Fig. Summary statistics of the gene expression profile of 9,835 samples in TCGA pan-cancer atlas.** (A) The density plots represent the sample distribution of observed z-scores of 10 genes. (B) The density plot and boxplot represent the sample distribution of observed z-scores of 16,323 genes. (C) The pie chart shows the proportion of the Pearson correlation values of 16,323 genes. All the expression values are log-transformed and z-transformed across cancers. The median expression values of samples across cancers are used for summary statistics.

**S2 Fig. Performance of our proposed method in four scenarios (I to IV) using naïve simulated data with the complete version.** (A) Synthetic data of correlated genes were generated from multivariate normal distribution with a mean value of 0 and a covariance matrix whose entries are 0.6. (B) Synthetic data of correlated genes were generated from multivariate normal distribution with a mean value of 0 and a covariance matrix whose entries are 0.3. The numbers in blue represent the average number of identified ceRNA-miRNA triplets after 100 simulations.

**S3 Fig. The performance of our proposed method in four scenarios (I to IV) using simulated data that mimics real-world data distribution with the complete version.** (A) From a covariance matrix with correlation values ranging from 0.3 to 0.9. (B) From a covariance matrix with correlation values ranging from 0.8 to 0.9. (C) From a covariance matrix with correlation values ranging from 0.5 to 0.7. (D) From a covariance matrix with correlation values ranging from 0.3 to 0.4.

- I could not find any open source license in the github repository. Please add one.
 We appreciate the comment and have added an open-source license to our Github repository ([https://github.com/ywhsiao/ceRNAR/blob/master/LICENSE.md](about:blank) and https://github.com/ywhsiao/ceRNAR/blob/master/DESCRIPTION).

#Minor:

- The manuscript states that "the partial correlation approach considers the expression of the shared miRNAs of two ceRNAs when calculating their correlation and ignores the interaction strength of them". I disagree here. Sensitivity correlation which is computed as the corrlation of two ceRNAs minus the partial correlation accounting for the miRNA which is referenced here as [14] certainly captures the effect size but albeit not the significance of an interaction. A measure of significance for sensitivity correlation was later suggested in 10.1093/bioinformatics/btz314.

We appreciate the suggestion and totally agree with the comment. We have removed this section.

- Figure 4: text is too small to read anything

We appreciate this comment and have divided this figure into four individual figures (S12-15 Fig and Fig 4) in the text and the supplementary section.

**Fig 4. An overview of our ceRNAR package.**

This package includes three main parts. First, “Input data” has three functions: *ceRNAputativePairs()* for extracting curated miRNA-target lists, *ceRNATCGA()* for retrieving TCGA data, and *ceRNAcustomize()* for importing customized data. Second, “Identification of miRNA-ceRNA triplets” involves *ceRNApairFiltering()* and *SegmentClusteringPlusPeakMerging()* and is wrapped into a *ceRNAmethod()* function. Lastly, “Downstream analyses” contains six functions for different tasks, that is, *ceRNAFunction()*, *ceRNAModule()*, *ceRNASurvival()*, *ceRNALocation(), ceRNAValidate() and ceRNAIntegrate()*.

**S12 Fig. Main tabular output in CSV format.** Five columns are involved: miRNA name, candidate ceRNA pairs, the start and end of each miRNA expression interval, and the number of segments.

**S13 Fig. The graphical bubble outputs of the *ceRNAFunctional* function are based on over-representation analysis (ORA).** (A) Based on the KEGG database. (B) Based on the ‘cellular component’ (CC) categories in the gene ontology (GO) database. (C) Based on the ‘molecular function’ (MF) categories in the GO database. (D) Based on the ‘biological process’ (BP) categories in the GO database.

**S14 Fig. The graphical bar outputs of the *ceRNAFunctional* function based on over-representation analysis (ORA).** (A) Based on the KEGG database. (B) Based on the gene ontology (GO) database and stratified by three GO levels: cellular component (CC), molecular function (MF), and biological process (BP).

**S15 Fig. The graphical outputs of the *ceRNAModule*, *ceRNASurvial*, and *ceRNALocation* functions.** (A) Network analysis among candidate ceRNAs for a specific miRNA. (B) Survival analysis for a candidate ceRNA pair targeted by a specific miRNA. (C) A mix of a box plot and bar plot represents the proportion of all candidate ceRNA pairs targeted by a specific miRNA at their corresponding miRNA expression range.
