# Supplementary material for: ceRNAR: An R package for identification and analysis of ceRNA-miRNA triplets
Source: PLoS Comput Biol. 2022 Sep 9;18(9):e1010497. doi: 10.1371/journal.pcbi.1010497 (PMC9491567; doi:10.1371/journal.pcbi.1010497)
Supplement: Attachment — Submitted filename: Reviewer_round2_final.docx [file pcbi.1010497.s035.docx]

Reviewer's Responses to Questions

**Comments to the Authors:
Please note here if the review is uploaded as an attachment.**

Reviewer #1: Authors succesfully answered my remarks.

We appreciate the constructive comments from reviewer #1.

Reviewer #2: The manuscript is significantly improved. In my opinion it is suitable for publication

We appreciate the constructive comments from reviewer #2.

Reviewer #3: The authors have done an admirable job improving the manuscript and in responding to the reviewer requests. Many of my previous questions have been answered. The underlying hypotheses are now much more clearly defined and the methods are better explained, especially through the figures that have been added. I would nevertheless recommend to include some of the explanations from the reviewer response into the manuscript, e.g. where the authors explained the intuition behind Pobey and Pviolate as well as the segment clustering since I believe this will make it much easier for readers to understand. The comparison with related tools that was now included offers a good overview of existing approaches. A few comments remain to be addressed:

We appreciate the suggestion and we have added the following paragraph into main manuscript to provide better explanation for the algorithm. Further comments are addressed point-by-point in the followings.

(line 477-490) “We have two assumptions in this study: (1) the expression levels of two target genes tend to be highly correlated when a possible ceRNA event occurs; (2) such events between target genes of a certain miRNA occur at a specific expression interval of that miRNA. However, for each ceRNA triplet from the real data, it is difficult to know which levels of miRNA expression (low, middle, or high expression intervals) will lead to a high correlation between a pair of target genes. Notably, for one miRNA, the high correlation values between two target genes can only be observed in a specific range of the miRNA expression. Definitely, the expression level of one miRNA can be regulated by many factors, such as compensation and/or other interactors. However, with our current understanding of all miRNAs, it is not feasible to consider all potential regulators of one specific miRNA at the same time. Therefore, for one single miRNA, we adapted the approach of utilizing its expression level as the final output instead of considering all possible confounding factors, which can be regarded as the hidden layers of the miRNA expression value.”

(line 505-510) “We adopted the sliding window approach to identify the correlation patterns of the two target genes within a specific range of expression values for the miRNA. That is the reason why we ranked the samples based on their miRNA expression value from each triplet to identify the correlation patterns and provided the number of samples that meet the criteria. Therefore, the …”

(line 527-540) “…[47], which captures the accumulated changes in the expression of all genes within a pathway through a random-walk method, to identify a significant triplet according to the number of the samples enriched in such an event. The main idea of the GSEA algorithm is to understand whether the differentially expressed genes are significantly enriched in the samples belonging to the same phenotype (case or control). First, the differentially expressed genes were ranked by using the differences in expression level between the two phenotypes. Next, the GSEA algorithm gave a positive score to the genes located in the pathway, whereas a negative score was assigned to the genes outside of the pathway. One possible scenario to obtain a high score is that the differentially expressed genes were clustered and enriched in one phenotype instead of being randomly distributed. This scenario is the same as what we want to identify for the ceRNA triplet. That is, the highest correlation values were observed in a specific range of the miRNA expression and thus we used the two statistics, P_obey_(k) and P_violate_(k) to identify the range (S11 Fig).”

(line 561-566) “For one miRNA, a low score S will be observed if the two target genes have no correlation within a specific range of miRNA expression. On the other hand, the score S will be high if a large proportion of the samples showed high correlation among the two target genes (P_obey_(k) is high). Consequently, we can use the score S to identify a ceRNA triplet when S is high, and a statistical approach was performed to ensure that S cannot be identified randomly.”

(line 569-579) “The motivation of the segment clustering is to group the samples showing high correlation between the expression levels of the two target genes into one single cluster. In our analysis, we divided the samples into small groups (i.e., a window) to calculate the correlation values of the two target genes. Accordingly, we may identify several different groups showing high correlations that actually can be clustered into one single cluster because of their similar correlation values. Therefore, … to evaluate whether those small windows showing high correlation values should be clustered into a larger group. This method has been widely used in the analysis of copy number variations (CNVs) by many algorithms [49, 50].”

(line 602-611) ”After performing the segment clustering approach, only a few groups of samples showing high correlation remain for further analyses. We have a basic assumption here for the ceRNA triplet: the correlation values should be stable across the sliding window approach and thus the correlation values should not be bouncing up and down within a small sample size. Following this assumption, we performed the peak merging step to ensure two nearby peaks were merged into one under certain criteria.”

- Figure S7: the caption does not explain what the legend labels mean. What is the meaning of no_validated, validated_both, validated_each?

We apologize for the confusion and have revised the descriptions for Figure S7.

**S7 Fig. Experimental validation of real case study based on miRSponge database.** (A) TCGA-LUAD (The Cancer Genome Atlas Lung Adenocarcinoma). (B) TCGA-LUSC (The Cancer Genome Atlas Lung Squamous Cell Carcinoma). Three different conditions are summarized here. ‘no_validated’ represents that no elements of the two mRNA-miRNA pairs identified by the ceRNAR package was reported as experimentally validated using the miRSponge database. ‘validated_both’ represents that both elements of the mRNA-miRNA pairs identified by the ceRNAR package were reported as experimentally validated using the miRSponge database. ‘validated_each’ represents that only one element of the mRNA-miRNA pairs identified by the ceRNAR package was experimentally validated using the miRSponge database.

- In a response to reviewer 1 ("Authors did not compare ceRNAR with other existing ceRNA predictors"), the authors claim that all five tested methods are correlation based. Just to clarify this I want to point out that mutual information and correlation are two distinct concepts, meaning that CUPID and JAMI which employ conditional mutual information can not be considered correlation-based methods.

We apologized for the mistake and have revised it. Excepting JAMI, the rest algorithms consider the correlations between miRNA-mediated genes/lncRNA.

(line 323-350) “All four of these algorithms are implemented in R. JAMI is based on conditional mutual information, which is particularly useful to capture non-linear associations by estimating the effect of a miRNA on its target pairs through a permutation test [26]. Excepting JAMI, the rest algorithms consider the correlations between miRNA-mediated genes/lncRNA. Although the majority of such … It is worth mentioning that CERNIA and JAMI consider both MRE- and expression-based data, whereas SPONGE, GDCRNATools and ceRNAR only analyze genome-wide expression data.”

- Table S17: While I really appreciate the comparison table for the different tools, I'd like to point out some issues. Some of the tested tools have no built-in "miRNA-targets data sources", thus it appears unsuitable to list specific data sources here. One should specify that the tools are flexible. For example saying "user-provided, e.g. miRecords…". The table probably needs a caption since some rows are unclear. For example, what is meant by the row "TCGA expression". Most tools are agnostic to the source of the expression data. Similarly, it is not fully clear what is meant by "case-only".

We appreciate the suggestions and have revised the display and contents of Table S17. (1) we have specified tools that have no built-in "miRNA-targets data sources". (2) We have coloured the background for even rows to make it easier to be read. (3) We have deleted this row that names “study design” to avoid confusion. Originally, this row was designed to show that these tools can accept the experiments results from a study that is not designed for case-control strategy.

(line321-323) “Notably, the four algorithms and our ceRNAR all used a similar strategy, which is utilizing miRNA-target data sources to identify potential miRNA-gene/lncRNA/pseudogene pairs from other databases.”

**S17 Table. Comparisons with the state-of-the-art tools.**

| **Features** | **ceRNAR** | **SPONGE** | **GDCRNATools** | **CERNIA** | **JAMI** | **Cupid** |
| --- | --- | --- | --- | --- | --- | --- |
| **miRNA-targets data sources** | miRTarBase, miRecords, DIANA-micro T-CDS, EIMMO, miRDB, miRanda, PITA, RNA22, TargetScan | User-provided e.g. TargetScan, miRcode, LncBase, mirTarBase | StarBase, miRcode,  miRTarBase, spongeScan | miRTarBase, starBase,  miRecords, miRanda, DT-Hybrid | miRecords, TarBase,  TRANSFAC, miRnada, PITA, Targetcan | miRecords, TarBase,  TRANSFAC, miRnada, PITA, Targetcan |
| **TCGA expression profile** | Yes | Yes | Yes | Yes | Yes | Yes |
| **ceRNA classes** | mRNA | mRNA, lncRNA | mRNA, lncRNA | mRNA,lncRNA, pseudogene | mRNA | mRNA |
| **ceRNA prediction algorithm** | Pearson correlation +  Data augmentation + Running sum statistics | Sensitivity correlation for  more than one miRNA | Hypergeometric test +  Pearson correlation +  Regulation similarity | MRE-based +  Gene-expression correlation +  SVM classification | Non-parameter + Multi-threading version of Cupid | MRE-based +  conditional mutual information |
| **P-value on ceRNA interaction score** | Yes | Yes | No | No | Yes | Yes |
| **Implementation** | R language | R language | R language | R language | Java, R language | Matlab |
| **Genome-wide screening** | Yes | Yes | Yes | Yes | Yes | Yes |
| **Reference** | N/A | M. List et al. 2019 | R. Li et al. 2018 | D. S. Sardina et al. 2017 | A. Hornakova et al. 2018 | H. Chiu et al. 2014 |

- The simulation experiment that is meant to show the performance of the various too-ls is based on a pre-built covariance matrix. This approach leads to linear relationships, giving correlation-based methods a decisive advantage here. The poor performance of JAMI can be attributed to this, as no non-linear relationships can be discovered here. However, simulating non-linear relationships is not realistically feasible and the authors should just discuss openly that this evaluation is unfair for JAMI.

We appreciate the suggestion and have added the following paragraph into “Discussion” section.

(line 408-414) “It is also worth mentioning that our simulation results were based on a predefined covariance matrix. That is the true positive events were from the correlation-based approaches, and thus such events only showed linear relationships among the elements. Notably, such design may lead to the poor performance of JAMI because their algorithm was developed by using the mutual information strategy, which was able to capture non-linear relationships among ceRNA pairs in addition to the linear ones.”

- Figure 3: The caption does not explain the different scenarios.

We appreciate the suggestion and have added them to the legend of Figure 3. Additionally, to make the label more consistent across the article, we changed the labels for different scenarios from ‘I to V’ (Roman numbers) to ‘1 to 5’ (Arabic numerals).

**Fig 3. Performance comparisons between ceRNIA, JAMI, SPONGE, GDCRNATools and ceRNAR packages using synthetic data that mimic the real-world case.** (A) Sensitivity and PPV of various tools at different correlation levels for 100 samples of 105 pairs of target genes under four scenarios (1 to 4). (B) Specificity and NPV of various tools at different correlation levels for 100 sample of 105 pairs of target genes under null scenario (scenario 5). “cor_levels” represents four different correlation levels (all: 0.3-0.9; high: 0.8-0.9; low: 0.3-0.4; mid: 0.5-0.7).
